# Rapamycin Alleviates Heart Failure Caused by Mitochondrial Dysfunction and SERCA Hypoactivity in *Syntaxin 12/13* Deficient Models

**DOI:** 10.1002/advs.202507210

**Published:** 2025-06-26

**Authors:** Run‐Zhou Yang, Fang Li, Jiao Liu, Shu‐Ang Li, Dan‐Hua Liu, Zhuanbin Wu, Pei‐Pei Liu, Wenju Liu, Bin Zhou, Cizhong Jiang, Haibing Zhang, Ying Yu, Jian‐Sheng Kang

**Affiliations:** ^1^ Clinical Systems Biology Laboratories The First Affiliated Hospital of Zhengzhou University Zhengzhou 450052 China; ^2^ Department of Anesthesiology Shanghai General Hospital Shanghai Jiao Tong University School of Medicine Shanghai 200000 China; ^3^ Department of Pharmacology and Tianjin Key Laboratory of Inflammation Biology School of Basic Medical Sciences Tianjin Medical University Tianjin 300070 China; ^4^ Department of Cardiology The First Affiliated Hospital of Zhengzhou University Zhengzhou 450052 China; ^5^ Replatium Inc. 55 YuanZhong Road, PuDong New Area Shanghai 201300 China; ^6^ School of Life Sciences and Technology Tongji University Shanghai 200092 China; ^7^ Shanghai Institute of Biochemistry and Cell Biology Center for Excellence in Molecular Cell Science Chinese Academy of Sciences Shanghai 200031 China; ^8^ Shanghai Institute of Nutrition and Health Chinese Academy of Sciences Shanghai 200031 China

**Keywords:** heart failure, mitochondria, rapamycin, SERCA, Syntaxin 12/13

## Abstract

SYNTAXIN 12/13 (STX12), a member of the syntaxin protein family enriched in the brain and heart, plays important roles in vesicle recycling. Currently, the role of STX12 in cardiovascular physiology remains unclear. Using zebrafish and mice, it is shown that STX12 loss leads to pericardial edema, cardiac malformations, and heart failure. *Stx12* depletion disrupts mitochondrial morphology, reduces iron and zinc levels, and impairs ATP production. *Stx12*‐deficient cardiomyocytes exhibit prolonged repolarization due to decreased sarcoplasmic reticulum Ca^2+^‐ATPase (SERCA) activity. Treatment with rapamycin, an mTOR inhibitor, restores mitochondrial protein expression and function by prompting the TFEB‐PGC1α axis, enhances SERCA activity via the CAMKII‐phospholamban pathway, and reduces the expression of stress markers. These findings suggest that STX12 plays an important role in the energy metabolism and metal homeostasis of cardiomyocytes. Enhancing mitochondrial function, autophagy, and SERCA activity through the administration of rapamycin may provide a potential therapeutic approach for cardiomyopathies associated with STX12 deficiency and hypometabolism.

## Graphical abstract

Rapamycin alleviates heart failure via TFEB and CaMKII pathways in Syntaxin 12/13 deficient models. *Stx12* deficiency causes heart failure via impaired iron trafficking to mitochondria, reducing respiratory complexes and sarcoplasmic reticulum Ca^2+^‐ATPase (SERCA). Rapamycin, an mTOR inhibitor, restores mitochondrial proteins via TFEB‐PGC1α and boosts SERCA activity via CaMKII‐phospholamban (PLB) pathway, alleviating hypertrophy. CDN1163, a SERCA activator, also rescues cardiac defects of *Stx12*‐deficient mice.

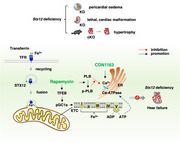

## 1 Introduction

Heart failure is a multifaceted clinical syndrome arising from various structural or functional cardiac disorders that impair the capability of pumping sufficient blood to meet the body's metabolic demands.^[^ ^1^ ^]^ It is a significant public health issue, with an estimated overall prevalence of 2.6% and a 50% 5‐year mortality rate.^[^ ^2^ ^]^ Common symptoms include dyspnea, fatigue, and exercise intolerance. Additionally, patients with heart failure are at higher risk for comorbidities like hypertension, diabetes, and obesity.

Cardiac hypertrophy, characterized by an increase in the size and mass of the heart muscle cells,^[^ ^3^ ^]^ is an adaptive response to various physiological or pathological stimuli, including stress, injury, or increased workload.^[^ ^4^ ^]^ In pathological hypertrophy, there is an increased expression of fetal genes, notably ANP (atrial natriuretic peptide), BNP (brain natriuretic peptide), β‐MHC (myosin heavy chain, a cardiac muscle β‐isoform), and skeletal muscle α‐actin. In contrast, these gene expressions remain normal or are decreased in physiological hypertrophy.^[^ ^5^ ^]^ Sustained pathological hypertrophy can ultimately lead to heart failure and increased mortality.^[^ ^3^ ^]^

SNARE (Soluble NSF Attachment Protein Receptor) proteins form a large family that is crucial for membrane fusion, a process vital for cellular functions such as exocytosis, endocytosis, and vesicular trafficking.^[^ ^6^ ^]^ These proteins regulate the fusion of vesicles with target membranes by forming a tight SNARE complex, composed of t‐SNAREs (target membrane SNAREs) and v‐SNAREs (vesicle SNAREs).^[^ ^7^ ^]^ Perturbations in SNARE proteins have been implicated in neurodegenerative disorders such as Parkinson's disease and Alzheimer's disease,^[^ ^8^ ^]^ as well as diabetes^[^ ^9^ ^]^ and cancer.^[^ ^10^ ^]^ In cardiac tissue, SNARE proteins play critical roles in regulating membrane ion channels^[^ ^11^ ^]^ or transporters.^[^ ^12^ ^]^ The deficiency of STX4 results in biventricular dilated cardiomyopathy or perinatal lethality, suggesting its important function in regulating normal embryonic cardiac function.^[^ ^13^ ^]^

STX12 is a member of the syntaxin family that is involved in various cellular processes, including melanosome biogenesis,^[^ ^14^ ^]^ platelet α‐granules,^[^ ^15^ ^]^ autophagosome maturation,^[^ ^16^ ^]^ ethanol preference,^[^ ^17^ ^]^ cellular invasion,^[^ ^18^ ^]^ and Fe^2+^ ion transport.^[^ ^19^ ^]^ It has been reported that STX12 localizes to endosomes and contributes to the recycling of surface receptors.^[^ ^20^ ^]^ Our previous works have demonstrated that *Stx12* knockout in mice leads to perinatal death with iron deficiency anemia,^[^ ^19^ ^]^ and revealed that STX12 is necessary in maintaining mitochondrial function, and STX12 depletion results in pulmonary mtDNA release and activates mtDNA‐dependent innate immunity.^[^ ^21^ ^]^ Here, we have explored the effects of *Stx12* deficiency on cardiovascular systems in zebrafish and mouse models. Our findings revealed that *Stx12* deficiency causes pericardial edema in zebrafish and cardiac malformation in mice. Conditional *Stx12* knockout in the heart results in cardiac hypertrophy and heart failure with cardiac mitochondrial morphological changes and energy deficiency. Moreover, cardiomyocytes lacking STX12 exhibit a prolonged decay phase of action potential, which is attributed to the reduced pumping activity of sarcoplasmic reticulum Ca^2+^‐ATPase (SERCA). Treatment with rapamycin, an mTOR inhibitor, activates autophagy, promotes SERCA activity via the phosphorylation phospholamban (PLB) by CAMKII, and enhances the expressions of mitochondrial respiratory complex proteins via prompting TFEB‐PGC1α pathway, effectively alleviating heart failure induced by STX12 deficiency.

## 2 Results

### 2.1 STX12 Deficiency Causes Cardiac Dysfunction

**Figure 1:**
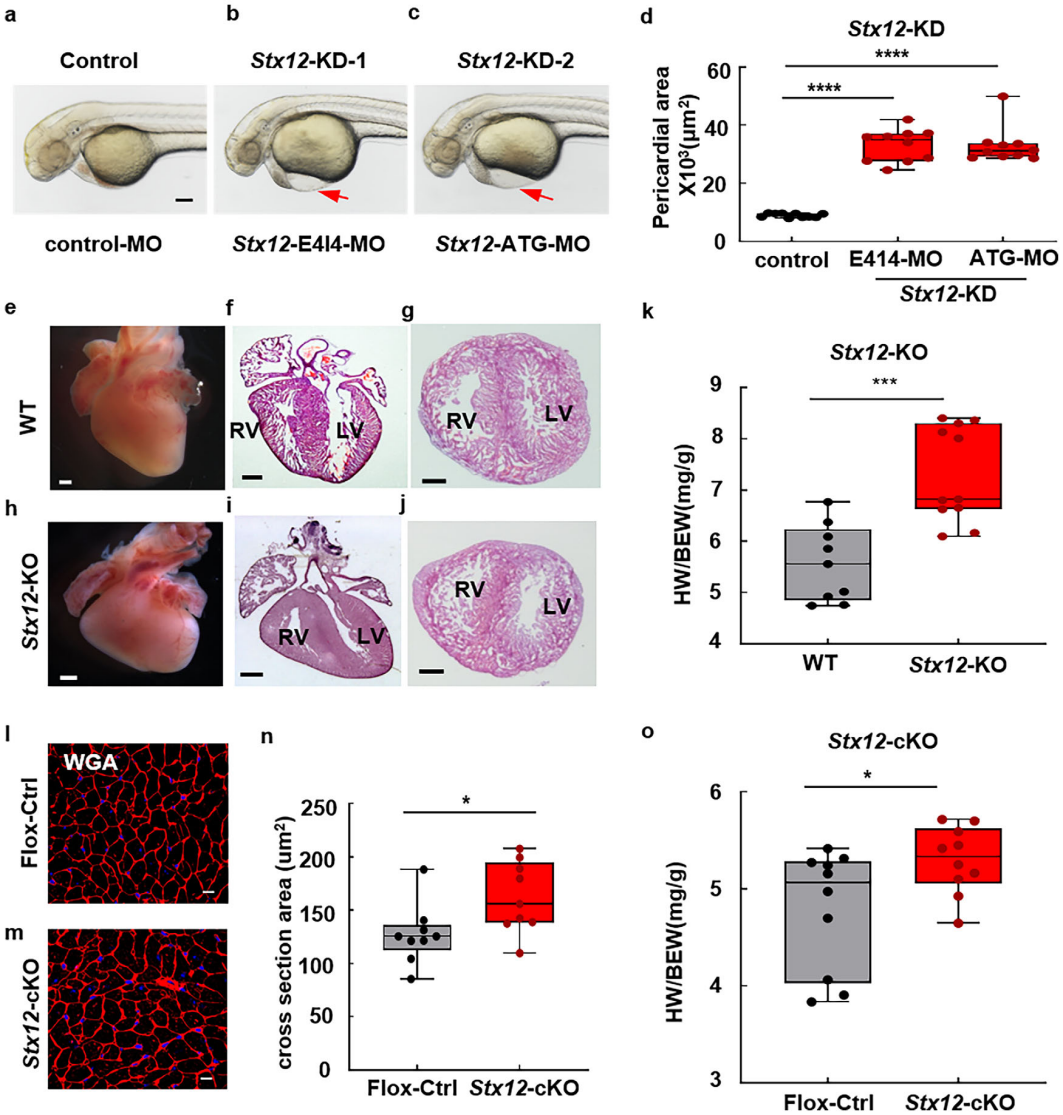
STX12 deficiency‐induced cardiac morphological changes in zebrafish and mice. a–c) Knockdown of *Stx12* in zebrafish led to pericardial edema. The phenotypes of zebrafish were represented at 2 dpf after injection of morpholinos into zebrafish eggs to knock down STX12 expression. Scale bar, 100 µm. d) Quantification of pericardial area. The pericardial area of zebrafish significantly increased after *Stx12* knockdown (control‐MO, *n* = 10, E414‐MO, *n* = 10*, t‐test, p *< 0.0001; ATG‐MO, *n* = 10, *p *< 0.0001). e,h) Cardiac stereomicroscopic images of P0 *Stx12*‐KO (e) and wild‐type littermates (h). *Stx12*‐KO heart exhibited cardiac malformation. Scale bar, 500 µm. f,g,i,j) Hematoxylin and eosin (HE) staining of coronal section (f, i) and cross section (g, j). Scale bars, 500 µm. k) Scatterplots with boxplots showed that heart weight‐to‐body weight ratio was significantly increased in *Stx12*‐KO mice (WT, *n* = 9, KO, *n* = 11, *p *= 0.0003). l,m) WGA staining of *Stx12* myocardial‐specific knockout mice and *Stx12*‐flox control mice. Scale bar, 10 µm. n) Quantitative analysis of WGA staining (Flox‐control, *n* = 9, cKO, *n* = 9; *p *= 0.0272). o) Scatterplots with boxplots showed that the heart weight‐to‐body weight ratio was significantly increased in myocardial‐specific *Stx12* knockout mice compared to *Stx12*‐flox control mice (Flox‐control, *n* = 10, cKO, *n* = 10, *p *= 0.0372). Statistical results: *p < 0.05, ***p < 0.001, ****p < 0.0001; *t‐test*.

Previous studies demonstrated that *Stx12* knockout in mice led to perinatal death with iron deficiency anemia,^[^ ^19^ ^]^ and resulted in pulmonary mtDNA release and mtDNA‐dependent innate immunity.^[^ ^21^ ^]^ To further investigate the physiological functions of STX12 in vivo, we knocked down *Stx12* (*Stx12*‐KD) in zebrafish by embryonic injection of morpholinos, a kind of modified nucleotides designed to specifically inhibit the translation of mRNA.^[^ ^22^ ^]^ Two types of morpholinos (Table S1, Supporting Information) were designed to either block the translation of the zebrafish gene (ATG‐MO) or affect the proper splicing of exon4 (E4I4‐MO) (Figure S1a,b, Supporting Information). In *Stx12‐*KD zebrafish injected with morpholino ATG‐MO or E4I4‐MO, prominent pericardial edema was observed at 2 dpf (day post fertilization) (**Figures** **1**a–c and S1c–e, Supporting Information). Quantitative analysis revealed a significant enlargement of the pericardial area in *Stx12*‐KD zebrafish (Figure 1d).

To explore the impact of *Stx12* deficiency on the mammalian cardiovascular system, we used the *Stx12* knockout (KO) C57/BL mouse strain,^[^ ^19^ ^]^ which was confirmed by western blotting analysis (Figure S2a, Supporting Information). These *Stx12*‐KO mice exhibited cardiac malformations, as evidenced by stereomicroscope and histological examination of heart tissue sections stained with hematoxylin and eosin (HE) (Figure 1e–j). The heart weight to body weight (HW/BW) ratio was significantly increased in the *Stx12*‐KO mouse (Figure 1k). However, wheat germ agglutinin (WGA) staining^[^ ^23^ ^]^ showed that the area of individual cardiac cell was smaller in *Stx12*‐KO mice compared to that of wild type (Figure S2b,c, Supporting Information). The elevated HW/BW ratio predominantly contributed to a reduced body weight of *Stx12*‐KO mice, a consequence of their perinatal lethality.

To further investigate the impact of STX12 deficiency specifically in the heart, we generated cardiac‐specific knockout mice (Stx12‐cKO) by crossing cTnT‐Cre mice with a conditional Stx12‐flox mouse line (*Stx12‐*flox). Mice with conditional *Stx12* knockout in the heart were not lethal but displayed signs of cardiac hypertrophy within three months. This was evidenced by an increase in the size of cardiac cells indicated by WGA staining (Figure 1l–n) and an elevated ratio of heart weight to body weight (HW/BW) (Figure 1o). Taken together, the presence of pericardial edema in *Stx12*‐deficient zebrafish, cardiac malformation in *Stx12*‐KO mice, and cardiac hypertrophy in conditional *Stx12* knockout mice consistently suggested that STX12 was crucial for maintaining normal cardiac function.

**Figure 2:**
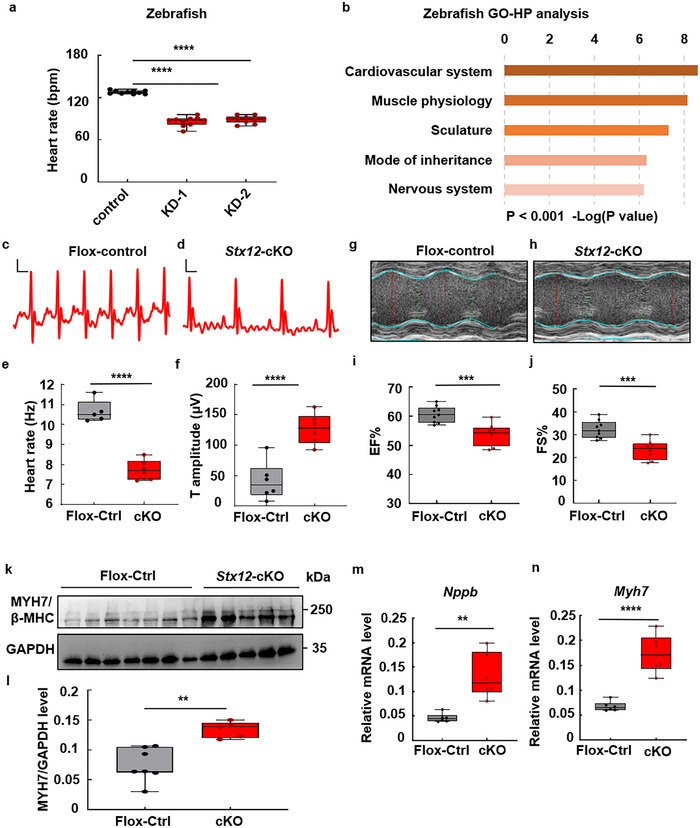
STX12 deficiency caused heart failure in zebrafish and mice. a) Quantification of the heart rates of zebrafish embryos. Heart rate was significantly decreased after *Stx12* knockdown (KD) in zebrafish (control, *n* = 10, KD‐1, *n* = 10, *t‐test, p *< 0.0001; KD‐2, *n* = 10, *p* < 0.0001). bpm, beats per minute. b) GO‐HP analysis of zebrafish RNAseq data suggested abnormalities of the cardiovascular system after *Stx12* knockdown. c,d) Electrocardiograms (ECG) of control (*Stx12*‐flox) (c) and cardiac‐specific *Stx12* knockout mice (d). Scale bars: horizontal 20 ms, vertical 100 µV. e) Quantification of heart rate derived from ECG. Heart rate in *Stx12* knockout mice was significantly decreased compared to wild type (Flox‐control, *n* = 5, CKO, *n* = 6, *p *< 0.0001). f) Comparison of T wave amplitude. T wave amplitude was significantly increased in *Stx12*‐cKO (*Stx12^flox/flox^* with *CTnT‐Cre*) mouse electrocardiograms compared to control (*Stx12^flox/flox^*) (Flox‐control, *n* = 6, cKO, *n* = 6, *p *<  0.0001). g,h) Representative M‐mode echocardiography of left ventricular chamber in control (*Stx12‐flox*) (g) and *Stx12* cardiac‐specific knockout mice (h). i,j) Measurement of ejection fraction (EF%) (i) and fractional shortening (FS%) (j) of *Stx12* cKO mice and control wild‐type mice. Left ventricular EF% in *Stx12*‐CKO mice was significantly decreased (Flox‐control, *n* = 9, cKO, *n* =  8, *p *= 0.0006). Left ventricular FS% in *Stx12*‐CKO mice significantly decreased (Flox‐control, *n* = 9, cKO, *n* = 8, *p *= 0.0004), indicating a decrease in ventricular contractile function. k) Western blot analysis of endogenous MYH7/β‐MHC level in control (*Stx12*‐flox) and *Stx12*‐cKO mice hearts. GAPDH was used as a loading control. l) Quantification of Western blot in (k). Myh7 level was significantly decreased compared with the wild type (Flox‐control, *n* = 7, cKO, *n* = 5, *p *= 0.0015). m,n) qRT‐PCR analysis of cardiac *Nppb* (m) and *Myh7* (n) mRNA levels in control (*Stx12*‐flox) and *Stx12*‐cKO mice heart (*n* = 6 mice per group). There were significant increases of the mRNA level of *Nppb* and *Myh7* in *Stx12*‐cKO mice (*Nppb*: Flox‐control, *n* = 6, cKO, *n* = 6, *p *= 0.0011; *Myh7*: Flox‐control, *n* = 6, cKO, *n* = 6, *p *< 0.0001), indicating myocardial hypertrophy and heart failure. Statistical results: **p < 0.01, ***p < 0.001, ****p < 0.0001; *t‐test*.

The pathophysiological effects of *Stx12* deficiency were further explored. *Stx12* deficiency led to a reduced heart rate in zebrafish (**Figure** **2**a and Movie S1, Supporting Information; data in mean ± standard deviation (SD): control, 127.9 ± 2.47 bmp; KD‐1, 86.2 ± 6.71 bmp; KD‐2, 88.9 ± 5.25 bmp; *t‐test*, *p* < 0.0001). Gene ontology (GO) analysis of zebrafish RNAseq data indicated that the cardiovascular and muscle systems of zebrafish were dramatically affected (Figure 2b). Moreover, electrocardiograph (ECG) recording in *Stx12* cardiac‐specific knockout mice (*Stx12*‐cKO) also displayed a reduced heart beating rate (Figure 2c–e; data in mean ± SD: cKO, 7.74 ± 0.49 Hz; control, 10.65 ± 0.56 Hz, *t‐test*, *p* < 0.0001) and an abnormal T wave with increased amplitude (Figure 2f). The T wave represents ventricular repolarization, which represents the recovery phase of the heart's electrical activity. An enlarged T wave in the ECG may suggest ventricular hypertrophy.^[^ ^24^ ^]^ M‐mode echocardiogram demonstrated that *Stx12*‐cKO mice exhibited significant decreases in ejection fraction (EF%) and fractional shortening (FS) in *Stx12*‐cKO mice compared to that of flox‐control (*Stx12‐*flox) mice (Figure 2g–j). A decreased EF% indicates reduced left ventricular function^[^ ^25^ ^]^ and heart failure. The flox‐control (*Stx12‐*flox) mice exhibited an EF% value of 61.2 ± 5.6% and a FS% value of 32.3 ± 3.9% (data in mean ± SD). In contrast, *Stx12*‐cKO mice had an EF% value of 47.4 ± 7.5% (*t‐test, p* = 0.0006), and an FS% value of 23.4 ± 4.2% (*t‐test, p* = 0.0004) (data in mean ± SD), indicating the occurrence of heart failure (Figure 2g,h,j).

To further confirm the heart failure phenotype in *Stx12*‐cKO mice at the molecular level, western blotting analysis was performed to investigate the expression of β‐myosin heavy chain (MYH7/β‐MHC), a marker associated with pathological remodeling in the heart. We found an upregulation of MYH7 in *Stx12*‐cKO mice (Figure 2k,l), providing extra evidence of heart failure. Consistently, the mRNA level of *MYH7* in *Stx12*‐cKO was also significantly increased compared to flox‐control (*Stx12‐*flox) mice (Figure 2n). In addition, natriuretic peptide B (NPPB), which serves as a prognostic indicator of heart failure,^[^ ^26^ ^]^ was significantly increased in transcriptional level (Figure 2m). Collectively, these findings substantiated that cardiac STX12 deficiency caused heart failure in mice.

### 2.2 STX12 Deficiency Impaired Mitochondrial Morphology and ATP Production

**Figure 3:**
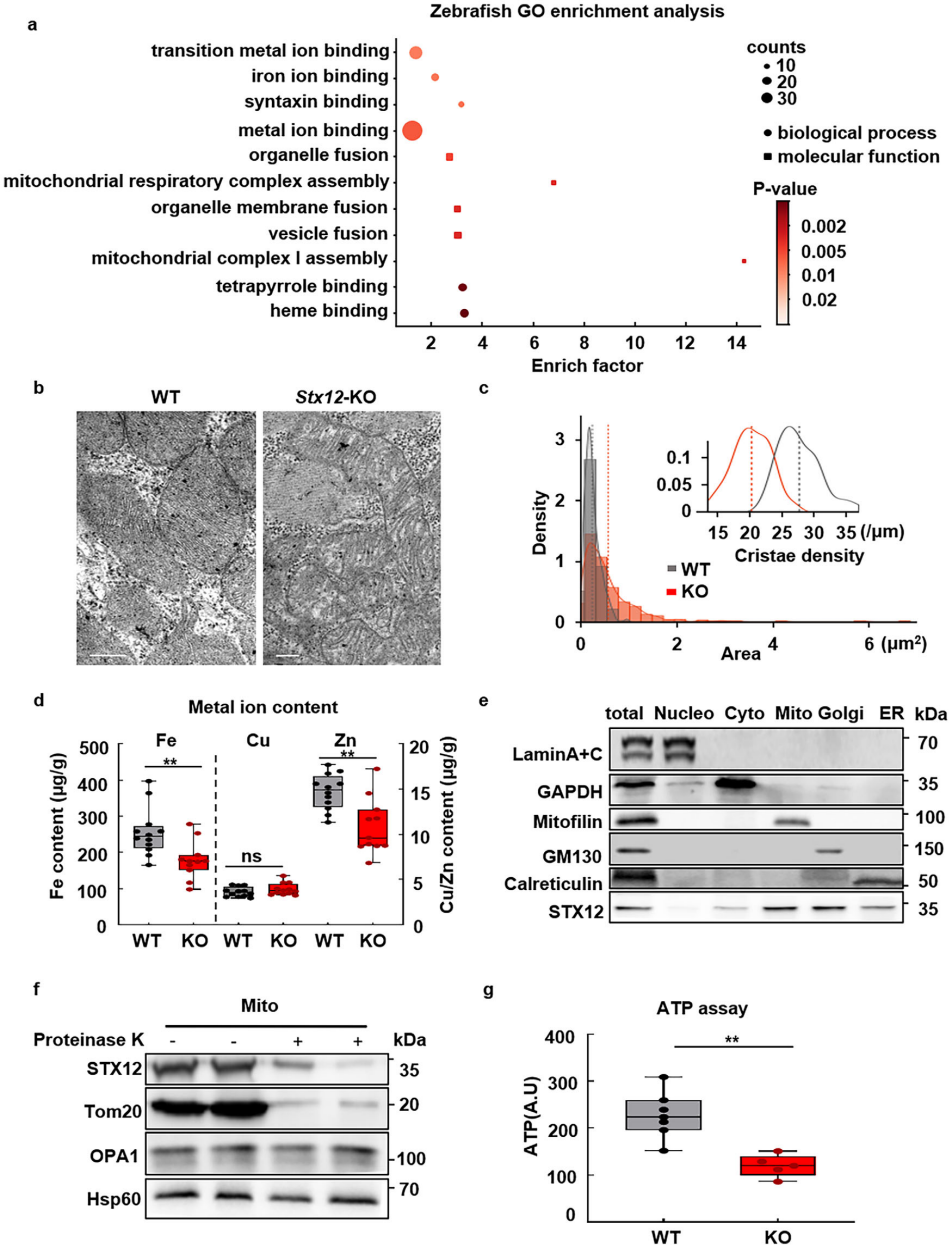
Abnormal mitochondrial morphology, ion imbalance, and energy deficiency in *Stx12* KO mouse. a) Zebrafish RNAseq GO enrichment analysis demonstrated the association of *Stx12* with iron binding, mitochondrial complex assembly, and vesicle fusion. b) Transmitting electron microscope images of heart sections in E18.5 wild type and *Stx12*‐KO mice. Representative EM image of heart section in *Stx12*‐KO (right), and wild type (WT) section (left). The scale bars represent 1 µm. c) Mitochondrial area and cristae density distribution of cardiac mitochondria. Mitochondria derived from *Stx12*‐KO (361 mitochondria from 3 KO) mice exhibited a significantly larger mitochondrial area than wild type (232 mitochondria from 2 WT). Inset showed that the cardiac mitochondria of *Stx12*‐KO (*n* = 57) had significantly decreased cristae density than the mitochondria of wild‐type (*n* = 57). Red dashed lines represented the averages (in mean ± SD) of *Stx12*‐KO mitochondrial area (0.61 ± 0.68 µm^2^) or cristae density (20 ± 3 µm^−1^), while gray dashed lines represented the mean of wild‐type mitochondrial area (0.28 ± 0.15 µm^2^, *t‐test, p* = 1.3 × 10^−17^) or cristae density (27 ± 3 µm^−1^, *p* = 1.7 × 10^−22^). d) Metal ion content in the heart tissues of *Stx12*‐KO and wild‐type littermate mice. Scatterplots with boxplots showed that the iron and zinc quantity in the hearts of *Stx12*‐KO pups significantly decreased compared to wild types (WT) (Fe: WT, *n* = 12, 255.97 ± 66.44 ug g^−1^, KO, *n* = 11, 178.91 ± 52.02 µg g^−1^, *p* = 0.0017; Zn: WT, *n* = 12, 14.72 ± 1.98 ug g^−1^，KO, *n* = 11, 10.89 ± 3.07 µg g^−1^, *p* = 0.0017), while the copper quantity remained unchanged (Cu: WT, *n* = 12, 3.95 ± 0.66 ug g^−1^, KO, *n* = 12, 3.95 ± 0.66 µg g^−1^, *p* = 0.2456). e) Western blot analysis revealed STX12 broad distribution in mitochondria, Golgi, and ER in cardiac tissue. f) STX12 is located in the outer membrane of mitochondria. Mitochondria extracted from cardiac tissue were treated with proteinase K, which digested surface proteins of mitochondria. Tom20, OPA1, and Hsp60 are located on the outer membrane, inner membrane, and matrix of mitochondria, respectively. After protein K treatment, OPA1 and Hsp60 expression remained unchanged, while Tom20 and STX12 expression decreased, indicating that STX12 was located on the outer membrane of mitochondria. g) ATP assay of P0 mouse homogenized heart tissue. ATP assay of homogenized mouse heart tissue. *Stx12* knock‐out cardiomyocyte exhibited a significantly decreased ATP level compared with normal mice. (WT, *n* = 7 mice; KO, *n* = 5 mice; *p* = 0.001183). Statistical results: **p < 0.01, ns, not significance; *t‐test*.

Zebrafish RNAseq gene ontology (GO) enrichment analysis revealed that STX12 deficiency was associated with iron ion binding and heme binding (**Figure** **3**a). In the genes associated with heme binding, such as hemoglobin (hbbe2 and hbbe1.1) was significantly downregulated (Figure S3, Supporting Information, hbbe2: log_2_FC (*Stx12*‐MO/control‐MO) = −1.02, −log_10_ *P* = 51; hbbe1.1:log_2_FC (*Stx12*‐MO/control‐MO) = −1.37, −log_10_ *P* = 106). This was consistent with our previous work that STX12 was involved in iron homeostasis in mice.^[^ ^19^ ^]^ Moreover, the RNA‐seq data also suggested a connection between *Stx12* and mitochondrial complex respiratory assembly (Figure 3a and Figure S3, Supporting Information).

The human heart is estimated to produce ≈6 kg of ATP daily.^[^ ^27^ ^]^ Mitochondria, as the cellular powerhouses, occupy about 30–40% volume of the cardiac muscle cells, generating 90% of total ATP through oxidative phosphorylation.^[^ ^28^ ^]^ Given the detrimental impact of STX12 deficiency on heart function, it was of particular interest to examine the potential effects on mitochondria and ATP production. Transmission electron microscopy analysis revealed abnormal mitochondrial morphology with reduced cristae density in E18.5 *Stx12*‐KO mice, as these mice were perinatal lethal (Figure 3b,c). As the GO enrichment analysis suggested STX12 was associated with metal ion homeostasis, metal ion contents of the heart of *Stx12*‐KO mouse were measured via inductively coupled plasma mass spectrometry (ICP‐MS) analysis. The results demonstrated that iron and zinc were significantly decreased while copper remained unchanged (data in mean ± SD: iron: WT: 255.97 ± 66.44 µg g^−1^, KO: 178.91 ± 52.02 µg g^−1^, *p* = 0.0057; zinc: WT: 14.72 ± 1.98 µg g^−1^, KO: 10.89 ± 3.07 µg g^−1^, *t‐test*, *p* = 0.0017; copper: WT: 3.65 ± 0.15 µg g^−1^, KO: 3.95 ± 0.19 µg g^−1^, *t‐test*, *p* = 0.2456) (Figure 3d and Table S2, Supporting Information).

As a member of the syntaxin protein family, STX12 is involved in intracellular vesicle fusion. The RNAseq GO enrichment analysis demonstrated that genes that participate in syntaxin binding and organelle membrane fusion were significantly affected (Figure 3a and Figure S3, Supporting Information). Intracellular subcellular fraction analysis with Western blot indicated that STX12 was colocalized with Golgi (GM130), endoplasmic reticulum (calreticulin), as well as mitochondria (mitofilin) (Figure 3e). Specifically, STX12 was located in mitochondrial outer membrane, which was evidenced by proteinase K digestion assay (Figure 3f). Moreover, in line with the crucial role of mitochondria in ATP production and the observed decrease in mitochondrial cristae density, ATP levels in *Stx12*‐KO mice were significantly reduced compared to wild‐type mice (*t‐test, p* = 0.0012) (Figure 3g). Taken together, these results demonstrated that STX12 had a role in metal homeostasis and was associated with mitochondria and energy production.

### 2.3 STX12 Deficiency Altered Cardiac Electrophysiology

**Figure 4:**
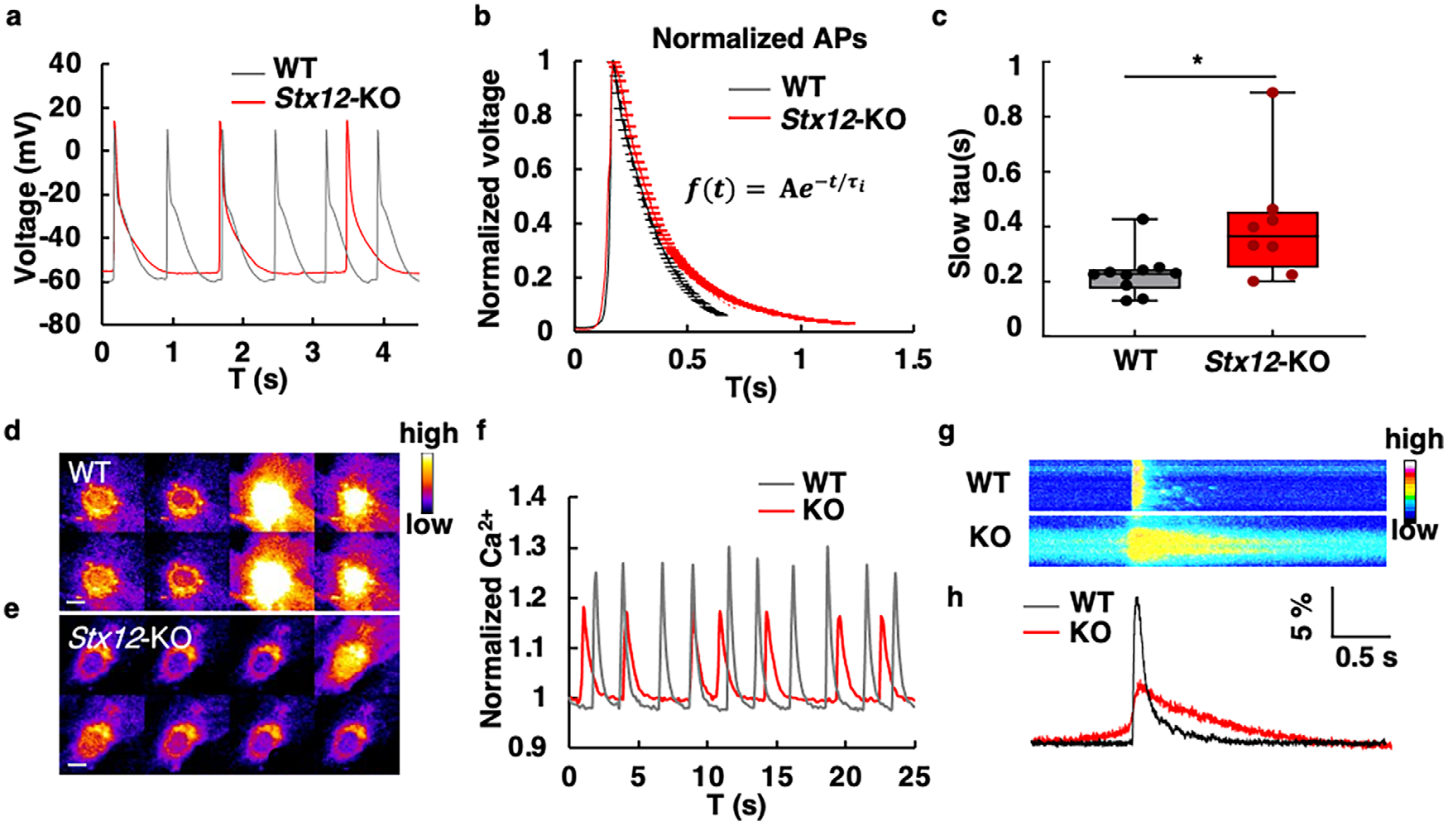
Abnormal electrophysiology of STX12 deficient cardiomyocytes. a) Patch‐clamp recordings of cardiomyocytes were primarily cultured from wild‐type (gray) and *Stx12*‐KO (red) mice. b) Normalized average action potential curves of wild‐type and *Stx12*‐KO mouse cardiomyocytes. The repolarization period was fitted with a single exponential decay curve (dashed line) (WT, black, 1361 action potentials of 9 cells; KO, red, 456 action potentials of 8 cells), and the decay constant (τ) was obtained from the fitted results. c) Comparison of the τ of the action potential curves (WT, black, *n* = 10 cells; KO, red, *n* = 8 cells, *t‐test*, *p* = 0.0272). d,e) Calcium imaging of wild‐type (d) and *Stx12*‐KO (e) mouse cardiomyocytes cultured in vitro. Images were represented as pseudo‐color, scale bars, 5 µm. The time interval between two images was 0.18 s. f) Calcium dynamics of wild‐type and *Stx12*‐KO mouse cardiomyocytes cultured in vitro. g) Representative calcium kymograph of single Ca^2+^ spike of wild‐type cardiomyocyte (upper) and *Stx12*‐KO (lower) cardiomyocyte. Images were represented as pseudo‐color. h) Calcium dynamical curve of single Ca^2+^ spike in (g). Compared to wild type, the peak value of *stx12*‐KO calcium concentration decreased while the decay phase time was prolonged. Statistical results: *p < 0.05; *t‐test*.

ATP is essential for both the contraction and relaxation of cardiac muscles, as it powers muscle contraction through actin and myosin filament sliding^[^ ^29^ ^]^ and facilitates muscle relaxation by driving calcium ion reuptake via sarcoplasmic reticulum pumps,^[^ ^30^ ^]^ thereby influencing voltage and calcium dynamics in cardiac cells. Thus, the lack of ATP in *Stx12* deficiency mice might affect the electrophysiology of cardiac cells. To investigate the effects of *Stx12* deficiency on cellular electrophysiology, the cardiomyocytes were isolated from *Stx12*‐KO mice. Despite the perinatal lethality of *Stx12*‐KO mice, the isolated cardiomyocytes were viable and exhibited continuous beating in vitro. Whole‐cell patch‐clamp recordings were performed in primary cultured *Stx12*‐KO and wild‐type cardiomyocytes to compare their electrical activities (**Figure** **4**a). Their action potentials (AP) were recorded and the decay phase of normalized action potentials was fitted with a single exponential function. The time constant tau (τ) was used to represent the decay rate of AP. The decay period of *Stx12*‐KO cardiomyocytes was prolonged compared to wild‐type cardiomyocytes (Figure 4b). The τ of *Stx12*‐KO cardiomyocytes was significantly increased compared to the τ of wild‐type cardiomyocytes, which was 0.40 ± 0.20 s in *Stx12*‐KO and 0.23 ± 0.077 s in WT (data in mean ± SD), respectively (*t‐test*, *p* = 0.0272) (Figure 4c). In addition, the calcium dynamics of *Stx12*‐KO cardiomyocytes were monitored with a calcium‐sensitive dye fluo‐4 using fluorescent microscopy (Figure 4d,e). The *Stx12*‐KO cardiomyocytes exhibited decreased frequency and amplitude with an increased decay period of calcium spikes (Figure 4f–h).

### 2.4 Reduced SERCA Activity in STX12‐Deficient Cardiomyocytes and Computational Modeling

**Figure 5:**
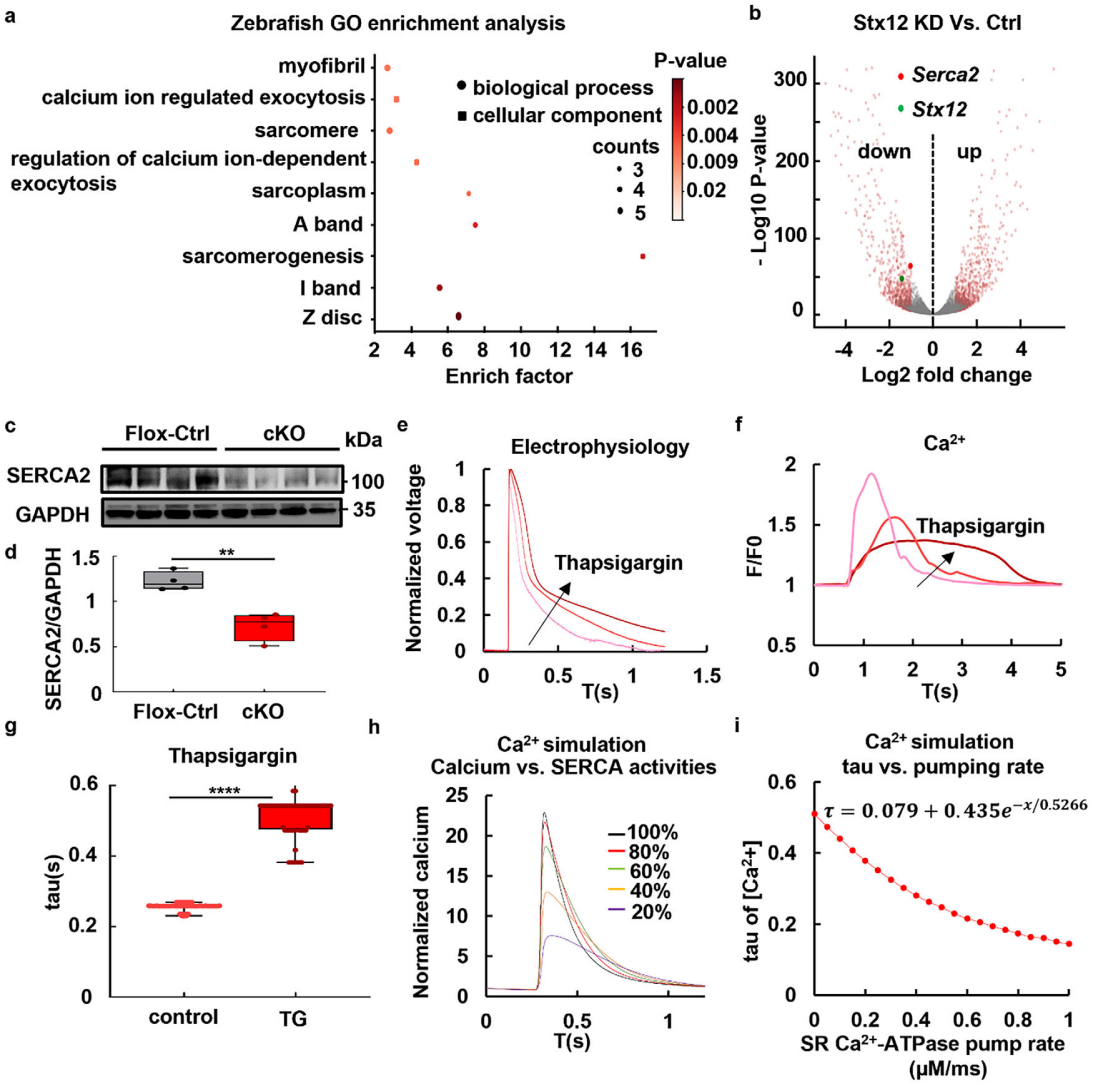
SERCA activity in STX12‐deficient cardiomyocytes and its role in calcium dynamics. a) Zebrafish RNAseq GO enrichment analysis revealed the association of STX12 with cardiac functions. b) Volcano plot of zebrafish RNAseq data. The genes that were significantly changed (*p* < 0.05) are marked with light red dots, while the rest were marked with gray dots. *Stx12* and the ortholog of *SERCA2* in zebrafish were marked with green and dark red dots, respectively. c) Western blot analysis of SERCA2 expression in control (*Stx12^flox/flox^*) and *Stx12*‐cKO (*Stx12^flox/flox^* with *CTnT‐Cre*) mice. d) Quantification of Western blot in (c). SERCA2 level was significantly decreased in *Stx12*‐cKO compared with control (*Stx12*‐flox) (Flox‐control, *n* = 4, cKO, *n* = 4, *p *= 0.0019). e) The normalized action potential curves of cardiomyocytes in vitro before and after adding thapsigargin (TG), with the arrow indicating the shift of the curve over time. f) The calcium dynamical curves with the arrow indicate the shift of the curve over time (0, 10, 20 min). g) The change in the decaying tau of Ca^2+^ in cardiomyocytes before and after adding TG (10 min). Tau was significantly increased after adding TG (control, *n* = 157 curves; TG, *n* = 72 curves, *t‐test, p* < 0.0001). h) The Ca^2+^ change curves under different SR Ca^2+^ pumps (SERCA) activities were obtained through model simulation. i) Curve of tau constants of Ca^2+^ during the decay phase of Ca^2+^ curves obtained through single exponential fitting under different SERCA Ca^2+^ pump activities. Statistical results: **p < 0.01, ****p < 0.0001; *t‐test*.

Zebrafish RNAseq gene ontology (GO) enrichment analysis demonstrated that *Stx12* was associated with sarcomere, sarcoplasm, and sarcomerogenesis (**Figure** **5**a), suggesting a vital role of *Stx12* in heart muscle cells. Genes associated with cardiac function were significantly affected (Figure S3, Supporting Information). Among these genes, the zebrafish ortholog of *Serca2*, *atp2a1*, was significantly downregulated (Figure 5b, log_2_FC (*stx12*‐MO/control‐MO) = −1.042, −log_10_ *P* = 62). Consistently, a reduced SERCA2 protein level was observed in cardiac *Stx12*‐cKO mice compared with flox‐control mice via western blotting (Figure 5c,d). As the repolarization period of action potential in cardiomyocytes involves the retrieval of calcium to intracellular calcium storage facilitated by SERCA, the prolonged action potential decay observed in STX12‐deficient cardiomyocytes (Figure 4b) might be attributed to decreased SERCA activity. As an ATP‐dependent enzyme, the activity of SERCA2 is determined by substrate concentration, enzyme concentration, potential post‐translational modifications, and the presence of inhibitors. To assess the impact of SERCA's activity on the decay phase of action potentials, we utilized thapsigargin (TG), an inhibitor of the SERCA pump. The addition of thapsigargin resulted in a time‐dependent delay in decay phases of action potentials observed via patch clamping recording in wild‐type cardiomyocytes (Figure 5e). Additionally, calcium imaging demonstrates that thapsigargin could widen the calcium curves, resulting in a dramatically increased tau (data in mean ± SD: WT, 0.26 ± 0.0087 s, TG, 0.53 ± 0.070 s, *p* < 0.0001) (Figure 5f,g). These findings demonstrated that decreased SERCA activity could lead to a prolonged decay and mimic the electrophysiological phenotype of STX12‐deficient cardiomyocytes. A computational model based on the Rasmusson^[^ ^31^ ^]^ model of cardiomyocytes was used to simulate this process. The parameter ν3 in Rasmusson's model, representing the maximum pump rate of SERCA, was adjusted to simulate calcium dynamics (Figure 5h,i and Supplementary Text, Supporting Information) and action potentials (Figure S4, Supporting Information). The tau‐pumping rate curve was well‐fitted with single exponential decay, yielding an *R*‐squared value of 0.9998 (Figure 5i). Collectively, the reduced SERCA activity in STX12‐deficient cardiomyocytes could affect the dynamics of the action potential, particularly prolonging the decay phase.

### 2.5 Rapamycin Alleviated Cardiac Dysfunction in STX12‐Deficient Cardiomyocytes

During cardiac hypertrophy, the heart undergoes increased synthesis of sarcomeric proteins, including actin, myosin, troponin, and tropomyosin. This process enhances the contractile capacity of cardiomyocytes but also consumes a significant portion of ATP, with protein biosynthesis consuming ≈33% of the total ATP expenditure.^[^ ^32^ ^]^ Rapamycin can reduce this ATP consumption by inhibiting the mammalian target of the rapamycin complex 1 (mTORC1) signaling pathway,^[^ ^33^ ^]^ which is overactivated in cardiac adaptation to the overload of pressure. Additionally, rapamycin promotes autophagy,^[^ ^34^ ^]^ which recycles cellular components and maintains ATP levels.

**Figure 6:**
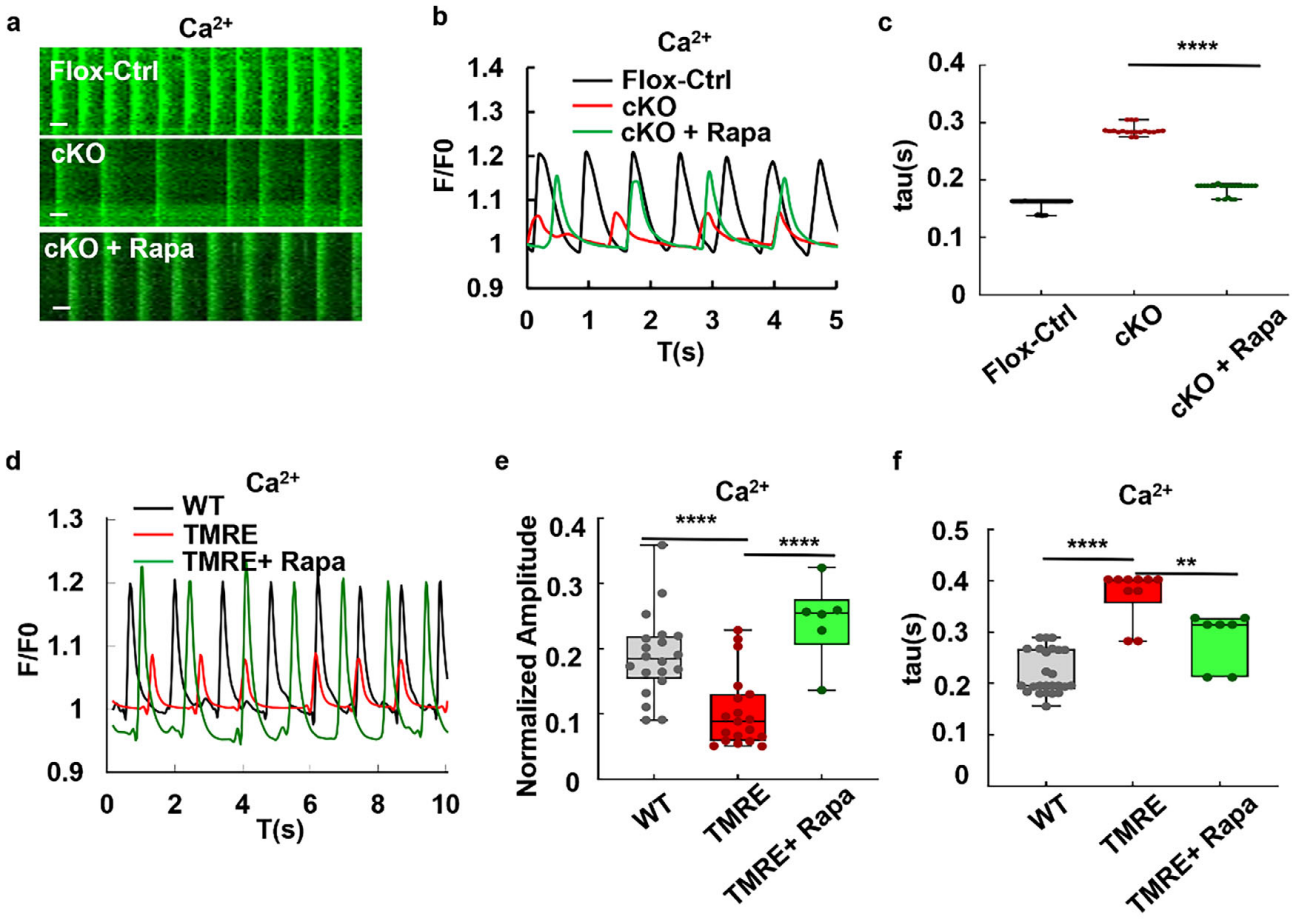
Rapamycin alleviated abnormal electrophysiology in *Stx12*‐cKO or energy‐deficient cardiomyocytes. a) Kymograph of Ca^2+^ dynamics in cultured control (*Stx12^flox/flox^*) cardiomyocytes, *Stx12*‐cKO (*Stx12^flox/flox^* with *CTnT‐Cre*) cardiomyocytes and *Stx12*‐cKO cardiomyocytes with rapamycin treatment. Time scale bars, 0.5 s. b) Calcium dynamical curves of control and *Stx12*‐cKO mouse cardiomyocytes cultured in vitro, before and after rapamycin administration. c) Decay constant of Ca^2+^ in *Stx12*‐CKO cardiomyocytes before and after rapamycin treatment. The decay constant of Ca^2+^ dynamics significantly decreased after rapamycin treatment (Flox‐control, *n* = 55 curves; CKO, *n* = 19 curves; cKO, rapamycin, *n* = 23 curves*; p* < 0.0001). d) Calcium dynamical curves of wild‐type cardiomyocytes in vitro with or without TMRE or rapamycin treatment (WT: black, TMRE: red, TMRE + rapamycin: green). e) Comparison of Ca^2+^ amplitudes in cardiomyocytes after TMRE and rapamycin treatment. The calcium amplitude was significantly reduced after TMRE treatment, and increased after rapamycin treatment (WT, *n* = 20; TMRE, *n* = 19, *p *< 0.0001; TMRE + rapamycin, *n* = 6, *p* < 0.0001). f) The decaying constant τ of Ca^2+^ in cardiomyocytes with or without TMRE or rapamycin treatment. Tau was significantly increased after TMRE treatment, and decreased after rapamycin treatment (WT, *n* = 16; TMRE, *n* = 10, *p* < 0.0001; TMRE + rapamycin, *n* = 7, *p *= 0.004). Statistical results: **p < 0.01, ****p < 0.0001; *t‐test*.

To explore the potential effects of rapamycin on cardiomyocyte dysfunction induced by STX12 deficiency, rapamycin was supplemented to a culture medium of isolated *Stx12*‐cKO cardiomyocytes. Strikingly, rapamycin treatment rescued the phenotype of calcium dynamics, where STX12 deficiency showed decreased frequency and amplitude (**Figure** **6**a,b), and an increased tau (Figure 6c) of calcium signals. In addition, action potential imaging with a cardiac promoter cTnT‐driven voltage indicator, SomArchon,^[^ ^35^ ^]^ also demonstrated that rapamycin could reverse the decreased frequency and the increased tau caused by STX12 deficiency (Figure S5, Supporting Information).

Tetramethylrhodamine ethyl ester (TMRE), a fluorescent dye accumulating in the mitochondrial matrix, can inhibit mitochondrial function at nano‐molar concentrations.^[^ ^36^, ^37^ ^]^ Therefore, TMRE was used to mimic the energy deficiency phenotype observed in STX12‐deficient cardiomyocytes. When cardiomyocytes were treated with TMRE at 50 nm concentration, the beating frequency revealed by calcium imaging in vitro was significantly decreased compared to control (mean ± SD: control, 1.11 ± 0.058 Hz; TMRE, 0.55 ± 0.094 Hz, *p* = 0.0006) (Figure 6d), and the calcium amplitude was also reduced (control, 18.8 ± 6.2%; TMRE, 10.4 ± 5.5%, *p* < 0.0001) (Figure 6e). Additionally, the decay tau was found to be increased (mean ± SD: control, 0.22 ± 0.041 s; TMRE, 0.37 ± 0.047 s, *p* < 0.0001) (Figure 6f). Consistently, rapamycin treatment could reverse the effects induced by TMRE, including the increased decay tau, the decreased frequency, and the reduced amplitude. Specifically, the combination of TMRE and rapamycin resulted in a restored frequency (TMRE + rapamycin, 1.14 ± 0.124 Hz, *t‐test* with TMRE, *p* = 0.0018), an increased calcium amplitude (TMRE + rapamycin, 24.3 ± 5.6%, *t‐test* with TMRE, *p* < 0.0001), and a shorter decay tau (TMRE + rapamycin, 0.29 ± 0.049 s, *t‐test* with TMRE, *p* = 0.004) (Figure 6e,f). These findings demonstrated that rapamycin treatment could effectively rescue abnormal electrophysiology not only in STX12‐deficient cardiomyocytes, but also in a hypometabolism model of wild‐type cardiomyocytes treated with TMRE.

The SERCA activity under various conditions could be estimated according to the tau‐pumping rate formula displayed in Figure 5i, and the results were summarized in Table S3 (Supporting Information). The simulation results suggested that STX12 deficiency could lead to a ≈53% decrease in SERCA pumping activity, while rapamycin could restore the pumping activity of SERCA to ≈81% of its maximum activity (Table S3, Supporting Information). 50 nm TMRE treatment might lead to a ≈77% decrease in SERCA pumping activity, which mimicked the phenotype of STX12‐deficient cardiomyocytes (Table S3, Supporting Information). Taken together, rapamycin treatment might partially rescue the abnormal electrical activity of STX12 deficiency or energy‐deficient cardiomyocytes via modulating SERCA activities.

### 2.6 Rapamycin Treatment Attenuated Heart Failure in STX12‐Deficient Mice

**Figure 7:**
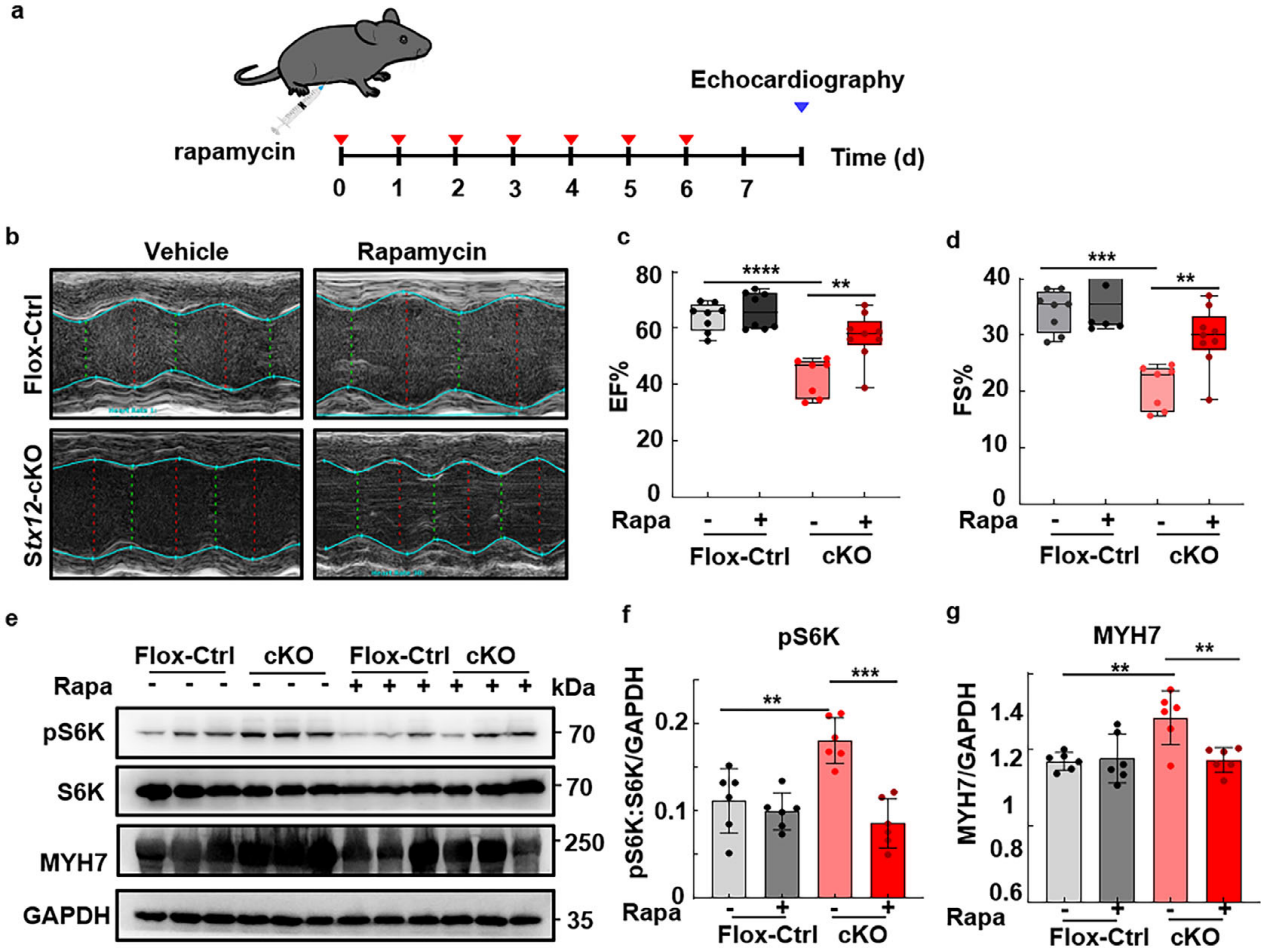
Rapamycin treatment relieved cardiac failure in *Stx12*‐cKO mice. a) Schematic diagram of rapamycin injection in mice. Cardiac functions were evaluated by echocardiography after intraperitoneally daily injection in *Stx12*‐cKO (*Stx12^flox/flox^* with *CTnT‐Cre*) and control (*Stx12^flox/flox^*) mice for one week. b) Representative M‐mode echocardiography of left ventricular chamber of control (*Stx12*‐flox) and *Stx12*‐cKO mice after rapamycin or vehicle treatment. c,d) Changes in ejection fraction (EF%) (c) and fractional shortening (FS%) (d) of the left ventricle in control (*Stx12*‐flox) and *Stx12*‐cKO mice after rapamycin or vehicle treatment. EF% was significantly increased in *Stx12*‐cKO mice after rapamycin treatment (Flox‐control + vehicle, *n* = 8; Flox‐control + rapamycin, *n* = 8; cKO + vehicle, *n* = 7, *t‐test, p *= 0.0003; cKO + rapamycin, *n* = 9, *p *= 0.0025). FS% was significantly increased in *Stx12*‐cKO mice after rapamycin treatment (Flox‐control + vehicle, *n* = 8; Flox‐control + rapamycin, *n* = 8; cKO + vehicle, *n* = 7, *p* = 0.0003; cKO + rapamycin, *n* = 9, *p *= 0.0025). e) Western blot analysis of S6K, phosphorylated S6K, and MYH7 in control (*Stx12*‐flox) and *Stx12*‐cKO mice, with GAPDH as the loading control. f) Quantification of Western blot of phosphorylated S6K (Flox‐control + vehicle, *n* = 6; Flox‐control + rapamycin, *n* = 6; cKO + vehicle, *n* = 6; cKO + rapamycin, *n* = 6; Flox‐control + vehicle vs cKO + vehicle, *p *= 0.00373; cKO + vehicle vs cKO + rapamycin, *p *= 0.0020). g) Quantification of Western blot of MYH7 (Flox‐control + vehicle, *n* = 6; Flox‐control + rapamycin, *n* = 6; cKO + vehicle, *n* = 6; cKO + rapamycin, *n* = 6; Flox‐control + vehicle vs cKO + vehicle, *p *= 0.0037; cKO + vehicle vs cKO + rapamycin, *p *= 0.0057). Statistical results: **p < 0.01, ***p < 0.001, ****p < 0.0001; *t‐test*.

Considering that rapamycin could effectively ameliorate malfunctions of cardiomyocytes caused by STX12 deficiency in vitro, we aimed to investigate the effectivity of rapamycin on alleviating heart failure in vivo. Rapamycin was injected intraperitoneally daily in both *Stx12*‐cKO and flox‐control (*Stx12*‐flox) mice for a week, and echocardiography was monitored subsequently (**Figure** **7**a). As anticipated, rapamycin demonstrated significant improvements in the reduced EF% and FS% caused by STX12 deficiency (data in mean ± SD: EF%: Flox‐control, 64.1 ± 5.05%; Flox‐control + rapamycin, 66.3 ± 6.52%; cKO, 42.5 ± 6.89%; cKO + rapamycin, 57.0 ± 8.46%, *t‐test* of cKO and cKO + rapamycin, *p* = 0.0025; FS%: Flox‐control, 34.2 ± 3.65%; Flox‐control + rapamycin, 36.0 ± 4.54%; cKO, 20.8 ± 3.93%, cKO + rapamycin, 29.7 ± 5.34%, *t‐test* of cKO and cKO + rapamycin, *p* = 0.0025) (Figure 7b–d). Moreover, the elevated T wave of ECG in *Stx12*‐cKO mice was also decreased upon rapamycin treatment (Figure S6, Supporting Information).

S6K, also named p70 ribosomal protein S6 kinase, plays a crucial role in cell growth, proliferation, and protein synthesis regulation. S6K phosphorylated by mTORC1,^[^ ^38^ ^]^ typically occurs in response to various cellular signals, including growth factors, hormones, and nutrients. In *Stx12*‐cKO mice, the level of phosphorylated S6K was increased compared to flox‐control (*Stx12*‐flox) mice, indicating that protein synthesis was enhanced. Administering rapamycin resulted in a decreased phosphorylation of S6K in *Stx12*‐cKO mice (Figure 7e,f), indicating an effective inhibition of mTORC1. Consistently, the therapeutic effect of rapamycin was demonstrated through its ability to suppress the overexpression of MYH7/β‐MHC induced by *Stx12*‐cKO in the heart (Figure 7e,g). Furthermore, rapamycin treatment reduced the mRNA levels of *Nppb* and skeletal α‐*actin*, which were elevated in *Stx12*‐cKO mice (Figure S7, Supporting Information).

### 2.7 Rapamycin Treatment Enhanced Mitochondrial Protein Synthesis and SERCA2 Activity

**Figure 8:**
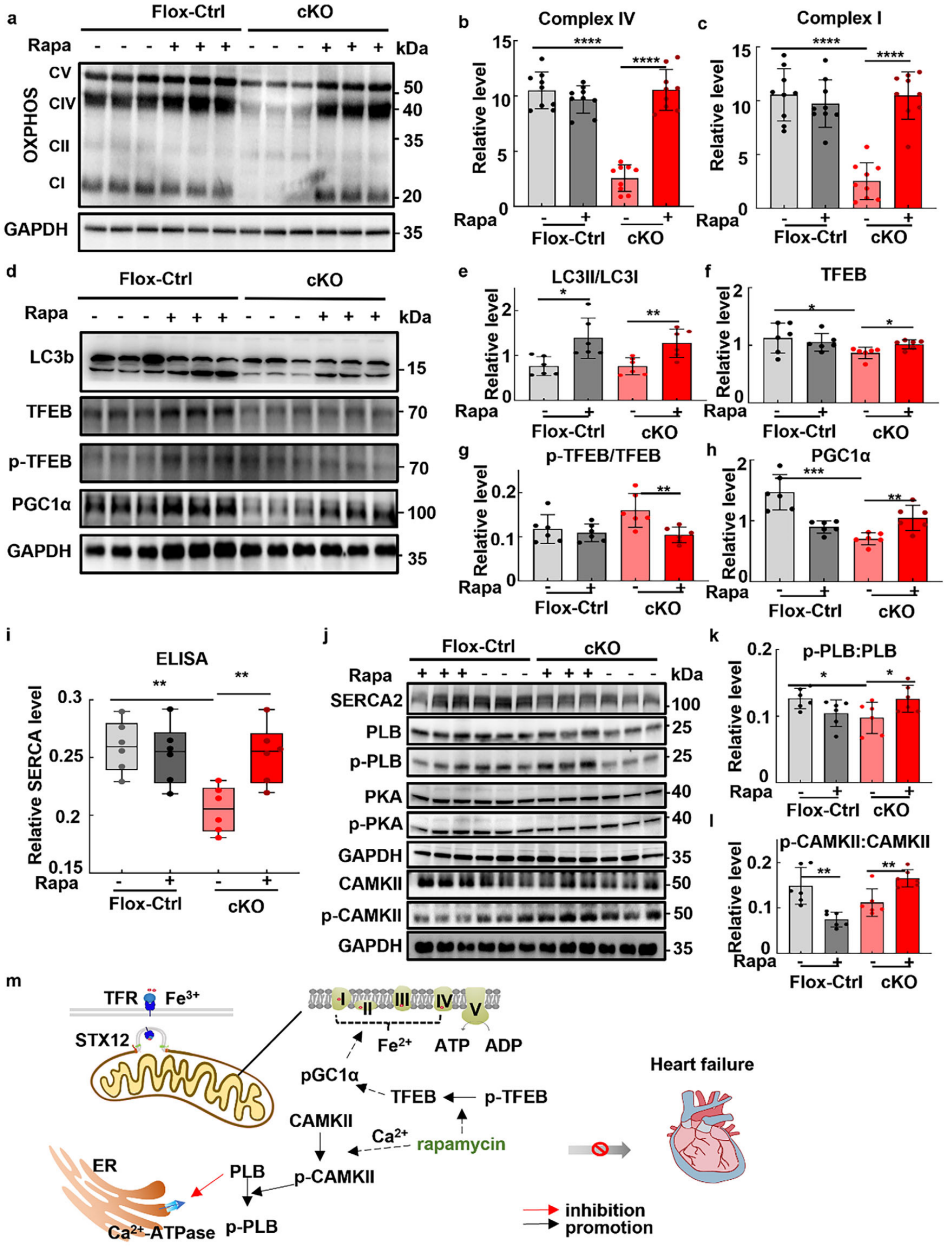
Rapamycin treatment enhanced mitochondrial protein synthesis and SERCA2 activity. a) Changes in the expressions of oxidative phosphorylation complexes (OXPHOS) in control (*Stx12*‐flox) and *Stx12*‐cKO mice after rapamycin or vehicle intervention, with GAPDH as the loading control. b) Quantification of Western blot of complex IV (Flox‐control + vehicle, *n* = 9; Flox‐control + rapamycin, *n* = 9; cKO + vehicle, *n* = 9; cKO + rapamycin, *n* = 9; Flox‐control + vehicle and cKO + vehicle, *p* < 0.0001; cKO + vehicle and cKO + rapamycin, *p* < 0.0001). c) Quantification of Western blot of complex I (Flox‐control + vehicle, *n* = 9; Flox‐control + rapamycin, *n* = 9; cKO + vehicle, *n* = 9; cKO + rapamycin, *n* = 9; Flox‐control + vehicle and cKO + vehicle, *p* < 0.0001; cKO + vehicle and cKO + rapamycin, *p* < 0.0001). d) Changes in the expressions of LC3b, TFEB, and its phosphorylated form as well as PGC1α in control (*Stx12*‐flox) and *Stx12*‐cKO mice after rapamycin or vehicle intervention, with GAPDH as the loading control. e) Quantification of Western blot of LC3II to LC3I ratio (Flox‐control + vehicle, *n* = 6; Flox‐control + rapamycin, *n* = 6; cKO + vehicle, *n* = 6; cKO + rapamycin, *n* = 6; Flox‐control + vehicle and Flox‐control + rapamycin, *p* = 0.0116; cKO + vehicle and cKO + rapamycin, *p* = 0.0068). f) Quantification of Western blot of TFEB (Flox‐control + vehicle, *n* = 6; Flox‐control + rapamycin, *n* = 6; cKO + vehicle, *n* = 6; cKO + rapamycin, *n* = 6; Flox‐control + vehicle and cKO + vehicle, *p *= 0.0463; cKO + vehicle and cKO + rapamycin, *p* = 0.0149). g) Quantification of Western blot of phosphorylated TFEB (Flox‐control + vehicle, *n* = 6; Flox‐control + rapamycin, *n* = 6; cKO + vehicle, *n* = 6; cKO + rapamycin, *n* = 6; cKO + vehicle and cKO + rapamycin, *p* = 0.0092). h) Quantification of Western blot of PGC1α (Flox‐control + vehicle, *n* = 6; Flox‐control + rapamycin, *n* = 6; cKO + vehicle, *n* = 6; cKO + rapamycin, *n* = 6; Flox‐control + vehicle and cKO + vehicle, *p* = 0.0001; cKO + vehicle and cKO + rapamycin, *p* = 0.0044). i) ELISA analysis of SERCA level in *Stx12*‐CKO (*Stx12^flox/flox^* with *CTnT‐Cre*) and control (*Stx12^flox/flox^*) mice after rapamycin or vehicle injection (Flox‐control + vehicle, *n* = 6; control + rapamycin, *n* = 6; cKO + vehicle, *n* = 6; cKO + rapamycin, *n* = 6; Flox‐control + vehicle and cKO + vehicle, *p* = 0.0012; cKO + vehicle and cKO + rapamycin, *p* = 0.0048). j) Western blot analysis of sarcoplasmic reticulum Ca^2+^ ATPase SERCA2, phospholamban (PLB) and its phosphorylated form, PKA and its phosphorylated form, as well as CAMKII and its phosphorylated form in control (*Stx12*‐flox) and *Stx12*‐cKO mice after rapamycin or vehicle intervention, with GAPDH as the loading control. k) Quantification of Western blot of phosphorylated phospholamban (Flox‐control + vehicle, *n* = 6; Flox‐control + rapamycin, *n* = 6; cKO + vehicle, *n* = 6; cKO + rapamycin, *n* = 6; Flox‐control + vehicle and cKO + vehicle, *p* = 0.0011; cKO + vehicle and cKO + rapamycin, *p* = 0.0019). l) Quantification of Western blot of phosphorylated CAMKII (Flox‐control + vehicle, *n* = 6; Flox‐control + rapamycin, *n* = 6; cKO + vehicle, *n* = 6; cKO + rapamycin, *n* = 6; Flox‐control + vehicle and Flox‐control + rapamycin, *p* = 0.0020; cKO + vehicle and cKO + rapamycin, *p* = 0.0042). m) A schematic shows that STX12 is involved in iron transportation and respiration complex assembly. Rapamycin treatment can enhance both mitochondrial protein biosynthesis and SERCA2 activity, ultimately contributing to the amelioration of heart failure in *Stx12*‐cKO mice. Statistical results: *p < 0.05, **p < 0.01, ***p < 0.001, ****p < 0.0001; *t‐test*.

Mitochondrial respiratory complexes, including five main complexes, are crucial for cellular energy production through oxidative phosphorylation. The expression of the mitochondrial respiratory complexes, particularly complex I, II, IV, and V, was decreased compared to wild type (**Figure** **8**a). Strikingly, rapamycin could significantly enhance the expression of the mitochondrial respiratory complexes of *Stx12*‐cKO mice, such as complex I and IV, in comparison to those treated with vehicle (Figure 8a–c). Rapamycin treatment resulted in an upregulation of the LC3B‐II to LC3B‐I ratio in both *Stx12*‐cKO and control mice (Figure 8d,e), indicating the activation of autophagy. TFEB, a master regulator of the autophagosome‐lysosome pathway, plays a pivotal role in cardiac hypertrophy.^[^ ^39^ ^]^ Its deficiency is linked to impaired autophagic flux, leading to the accumulation of damaged organelles and protein aggregates in the hypertrophic process. Phosphorylated TFEB remains cytoplasmic, whereas dephosphorylation facilitates nuclear translocation, enabling gene activation, including peroxisome proliferator‐activated receptor gamma coactivator‐1α (PGC‐1α) and TFEB itself.^[^ ^40^ ^]^ Western blotting demonstrated that TFEB level was decreased in *Stx12*‐cKO mice compared to control (*Stx12*‐flox) mice (Figure 8d,f). TFEB can be phosphorylated by multiple kinases, including mTORC1, protein kinase Cβ (PKCβ), extracellular signal‐regulated kinase 2 (ERK2), AKT serine/threonine kinase (Akt), glycogen synthase kinase 3 beta (GSK3β), and mitogen‐activated protein kinase 3 (MAP4K3).^[^ ^40^, ^41^ ^]^ Consequently, rapamycin inhibiting mTOR signaling can partially and significantly decrease the phosphorylation of TFEB in *Stx12*‐cKO mice (Figure 8d,g). PGC1α (peroxisome proliferator‐activated receptor gamma coactivator 1‐alpha) is a protein that plays a crucial role in regulating cellular energy metabolism and mitochondrial biogenesis.^[^ ^42^ ^]^ Western blotting demonstrated that the expression of PGC1α was increased upon rapamycin treatment in *Stx12*‐cKO mice (Figure 8d,h). This finding suggested a beneficial role for rapamycin in enhancing mitochondrial function via promoting PGC1α expression through TFEB dephosphorylation. The mTOR signaling is known to regulate mitochondrial activity and biogenesis.^[^ ^43^ ^]^ Intriguingly, inhibiting overactivated mTOR signaling with rapamycin could alleviate cardiac dysfunction in *Stx12*‐deficient mice partially via enhancing mitochondrial protein synthesis. The finding suggested that the relationship between mTOR signaling and mitochondrial biogenesis was complicated rather than a monotonical correlation.

Furthermore, rapamycin treatment could increase the expression of SERCA2 in *Stx12*‐cKO mice compared to flox‐control (*Stx12*‐flox) mice, which were verified by an enzyme‐linked immunosorbent assay (ELISA) and western blotting (Figure 8i,j). Phospholamban, in its unphosphorylated state, acts as a regulatory protein that interacts directly with SERCA2 and inhibits its activity to pump calcium into the sarcoplasmic reticulum. Phosphorylation of PLB attenuates this inhibitory effect, thereby potentiating the calcium‐pumping function of SERCA2 and facilitating muscle relaxation.^[^ ^44^, ^45^ ^]^ The level of phosphorylated phospholamban was decreased in *Stx12*‐cKO mice compared to control (*Stx12*‐flox) mice, indicating the inhibition of SERCA2 activity in *Stx12*‐cKO mice. Upon rapamycin treatment, the level of phosphorylated PLB was increased compared to those treated with vehicle in *Stx12*‐cKO mice (Figure 8j,k), suggesting that rapamycin could also regulate the activities of SERCA2 via promoting PLB phosphorylation. The phosphorylation of phospholamban (PLB) involves several kinases and signaling pathways, including the protein kinase A (PKA) pathway and the calcium/calmodulin‐dependent protein kinase II (CaMKII) pathway. To further explore the mechanism of PLB phosphorylation, the level of phosphorylated‐PKA and phosphorylated‐CaMKII was compared via Western blotting. The results showed that while the level of phosphorylated‐PKA remained unchanged, the level of phosphorylated‐CaMKII was elevated upon rapamycin treatment in *Stx12*‐cKO mice (Figure 8j,l). The findings demonstrated that CaMKII was responsible for the phosphorylation of PLB upon rapamycin treatment in *Stx12*‐cKO mice. Since STX12 deficiency causes the decreased activity of SERCA2a, enhancing SERCA2a activity could be a viable therapeutic strategy for STX12‐related cardiomyopathies. Drugs that directly enhance SERCA's activity might alleviate the cardiac dysfunction in *Stx12*‐cKO mice. CDN1163, a small molecule activator of SERCA, has shown promise in modulating cellular functions related to diabetes and metabolic disorders.^[^ ^46^ ^]^ Here, CDN1163 treatment significantly increased EF% and FS% in both *Stx12*‐cKO mice and wild‐type mice (Figure S8, Supporting Information), demonstrating the therapeutic potential of targeting SERCA activity to ameliorate cardiac dysfunction. Taken together, rapamycin treatment could enhance both mitochondrial protein biosynthesis and SERCA2a activation, ultimately contributing to the amelioration of heart failure in *Stx12*‐cKO mice (Figure 8m).

## 3 Discussion

Our previous studies have shown that STX12 deficiency is neonatally lethal and affects the recycling of TFR receptors,^[^ ^19^ ^]^ that STX12 is necessary for maintaining mitochondrial function, and that STX12 depletion results in pulmonary mtDNA release activating mtDNA‐dependent innate immunity.^[^ ^21^ ^]^ In this study, we investigated the role of STX12 in the cardiovascular system and found that STX12 deficiency led to heart failure in both zebrafish and mice, indicating an evolutionarily conserved role in cardiovascular physiology. A key discovery is that STX12 deficiency disturbs mitochondrial morphology, metal homeostasis, ATP production, respiratory complexes, and SERCA activity in the heart. Although early studies have demonstrated that STX12 is located in endosomes, we now have found that a large portion of STX12 is located on the mitochondrial outer membrane. Given that STX12 is strongly colocalized with TFR vesicles,^[^ ^19^ ^]^ STX12 might be responsible for iron transportation to mitochondria. Iron, as an indispensable element of iron‐sulfur clusters and hemoproteins in mitochondria respiratory chain complexes I, II, III, and IV, is essential for the assembly and function of the electron transport chain (ETC).^[^ ^47^ ^]^ Consequently, our findings demonstrate that the protein levels of complex I, and IV are decreased in cardiac‐specific *Stx12* knockout mice (Figure 8a). Our findings support the hypothesis that STX12 facilitates the transport of iron from TFR‐containing vesicles to mitochondria, thus contributing to the assembly of respiratory complexes (Figure 8m).

The ECG of cardiac‐specific *Stx12*‐deficient mice has shown an increased T wave, indicating abnormal ventricular repolarization. This is further confirmed by electrophysiology and calcium imaging in primary cultured *Stx12*‐deficient cardiomyocytes. The prolonged decay phase of action potential in *Stx12*‐deficient cardiomyocytes is attributed to the decreased SERCA activity. In contrast, in *Stx4*‐deficient zebrafish, there is a decrease in ventricle calcium duration and a reduced activity of the L‐type Ca^2+^ calcium channel, rather than SERCA.^[^ ^13^ ^]^ During cardiomyocyte relaxation, SERCA utilizes ATP to pump Ca^2+^ ions from the cytoplasm into the sarcoplasmic reticulum, lowering intracellular calcium concentration and facilitating the preparation of heart muscle for subsequent contraction. Directly inhibiting SERCA by thapsigargin results in a prolonged decay phase of action potential in cardiomyocytes. The computational model of cardiomyocytes also supports that reducing SERCA activity can increase the decay tau in both calcium dynamic and action potential. A decrease in SERCA activity is strongly associated with the development and progression of heart failure.^[^ ^48^, ^49^, ^50^ ^]^ Therefore, drugs that enhance SERCA2a activity such as enalapril, oxymatrine, and etomoxir, as well as SERCA2 viral gene delivery methods have been used to improve cardiac function and treat heart failure.^[^ ^48^, ^49^, ^50^ ^]^ Indeed, CDN1163, an activator of SERCA, significantly mitigated cardiac dysfunction in cardiac‐specific *Stx12*‐deficient mice (Figure S8, Supporting Information).

Mitochondrial dysfunction and SERCA hypoactivity likely account for many of the pathological phenotypes observed in *Stx12*‐deficient hearts, including cardiomyocyte hypertrophy, decreased contraction, and prolonged repolarization phase. This is supported by the results of TMRE, which inhibits mitochondrial function and partially recapitulates the phenotype of *Stx12*‐deficient cardiomyocytes. During cardiac hypertrophy, there is a notable metabolic shift from fatty acid oxidation to increased glucose utilization, resulting in metabolic remodeling.^[^ ^51^ ^]^ Interestingly, although no cardiac hypertrophy was observed in *Stx12*‐KO heart as indicated by WGA staining (Figure S2b,c, Supporting Information), there is a prominent decrease of ATP in *Stx12*‐KO mice (Figure 3), which cannot survive for more than 12 h after birth.^[^ ^19^ ^]^ Our findings suggest cardiac hypertrophy is an adaptive process that typically occurs gradually over time, so that *Stx12*‐KO mice did not have sufficient time to develop hypertrophy.

Rapamycin exhibits protective effects on *Stx12*‐deficient cardiomyocytes in vitro and cardiac‐specific *Stx12*‐cKO mice in vivo. Previous studies have demonstrated that rapamycin has several protective effects on the heart,^[^ ^33^ ^]^ including reducing aortic banding‐induced cardiac hypertrophy,^[^ ^52^, ^53^ ^]^ improving postinfarction heart failure,^[^ ^54^ ^]^ persistently enhancing diastolic function and myocardial stiffness,^[^ ^55^ ^]^ as well as reversing age‐related heart dysfunction.^[^ ^56^ ^]^ In aortic banding‐induced hypertrophy, rapamycin partially reversed the increased expression of ANP, BNP, α‐skeletal actin, and β‐MHC, as well as the decreased levels of α‐MHC and SERCA2a.^[^ ^48^ ^]^ Besides these effects of rapamycin, we observed that rapamycin could also promote mitochondrial biogenesis via the TFEB‐PGC1α‐mitochondrial biogenesis axis and the phosphorylation of PLB in *Stx12*‐cKO mice, thus enhancing the activity of SERCA2a. Furthermore, rapamycin‐induced phosphorylation of PLB in *Stx12*‐cKO mice involved the calcium/calmodulin‐dependent protein kinase II (CaMKII) pathway, which was consistent with the elevated calcium level in rapamycin‐treated STX12‐deficiency cardiomyocytes (Figure 6).

Notably, administering 2.5 mg kg^−1^ of rapamycin intraperitoneally enhanced mitochondrial complex expression in our experiments (Figure 8a). In contrast, higher doses (≈8 mg kg^−1^) of rapamycin application in a Ndufs4 knockout mouse model of Leigh syndrome mitigated mitochondrial disease symptoms without affecting mitochondrial complex expression.^[^ ^57^ ^]^ Moreover, doses exceeding 8 mg kg^−1^ were found to be more beneficial for health and survival in the Ndufs4 knockout model.^[^ ^58^ ^]^ Therefore, the dose‐dependent effects of rapamycin on various disease models warrant further detailed investigation.

In summary, our findings have demonstrated that STX12 deficiency may hinder iron transportation to mitochondria, impairing mitochondrial morphology and function, causing hypometabolism and metabolic reprogramming, decreasing the activity of SERCA, and eventually resulting in heart failure. Therefore, the cardiac *Stx12*‐cKO mouse is a valuable model for intervening in hypometabolism of heart failure. Rapamycin intervention effectively alleviates heart failure symptoms caused by STX12 deficiency through mechanisms involving augmented autophagy, rejuvenation of mitochondrial function, and restoration of SERCA activity (Figure 8m). This multifaceted therapeutic potential of rapamycin intervention provides a promising strategy for combating diseases characterized by mitochondrial dysregulation or impaired energy metabolism, as exemplified in various forms of cardiomyopathy.

## 4 Experimental Section

### Plasmid Construct

SomArchon was obtained from Addgene (Addgene ID 126941). For expression in cardiomyocytes, the cTnT promotor (−1 to −589) was amplified from mouse genome DNA. For AAV virus packaging, SomArchon with promotor was cloned into the pAAV‐MCS vector.

### Gene Formatting

Standard gene formatting was used^[^ ^59^ ^]^: italicized initial uppercase followed by lowercase letters for rodents (*Stx12*). For the formal names of proteins, the names in nonitalicized uppercase letters (STX12) are used.

### RNAseq Data Processing

The total RNA of zebrafish was extracted using Trizol at 2 dpf after morpholino injection. The sequencing library was built using the Illumina TruseqTM RNA sample prep kit. Raw RNA sequences of zebrafish or mouse samples were sequenced by Novogene Co. Ltd. Cutadapt (V1.18) was used to remove adaptor sequences, low‐quality bases, and reads shorter than 50 bases with parameters “–a AGATCGGAAGAGC–AAGATCGGAAGAGC–trim‐n‐m 50‐q 20,20.” Next, the trimmed clean data were mapped to the mm10 reference genome using the Hisat2 (V2.1.0) with parameters “–dta‐cufflinks–no‐discordant.” After that, gene expression levels were quantified as Fragments Per Kilobase of exon model per Million mapped fragments (FPKM) by stringtie (V1.3.4d). Genes with FPKM < 1 in all samples were filtered, and FPKM values of replicates were averaged. Gene expression comparisons between *the Stx12*‐KO sample and the WT sample were performed by cufflinks (V1.3.0) with default parameters. Then, the significant differentially expressed genes (DEGs) were selected with a *p*‐value less than 0.05.

### RNAseq Gene Ontology (GO) Analyses

DEGs of zebrafish samples were further filtered using absolute values of log2(FC, fold change) and *p*‐values adjusted with the Benjamini‐Hochberg method. Then, ≈300–1000 top filtered DEGs were used for GO analyses via the online function profiling of g:Profiler (https://biit.cs.ut.ee/gprofiler/gost), such as Molecular Functions (MF), Biological Process (BP), Cellular component (CC), KEGG, Reactome (REAC), WikiPathways (WK), and Human Phenotype Ontology (HP).^[^ ^60^ ^]^

### Zebrafish Maintenance

Adult zebrafish were maintained at 28.5 °C on a 14 h light/10 h dark cycle. Five to six pairs of zebrafish were set up for natural mating every time. Embryos were maintained at 28.5 °C in fish water (0.2% Instant Ocean Salt in deionized water).

### Zebrafish Microinjections

GeneTools, LLC (http://www.genetools.com/) was used to design the morpholino (MO). Antisense MOs (GeneTools) were microinjected into fertilized one‐cell stage embryos according to standard protocols.^[^ ^61^ ^]^ The sequences of the stx12 translation‐blocking and splice‐blocking morpholinos were 5′‐TGGAGCAAACTACAGCAGGAAGCCA‐3′(ATG‐MO) and 5′‐ACTGGCAACTACAAAAGTACCTGTT‐3′(E4I4‐MO), respectively. The sequence for the standard control morpholino was 5′‐CCTCTTACCTCAGTTACAATTTATA‐3′(GeneTools). The amount of the MOs used for injection was as follows: Control‐MOandE4I4‐MO,4 ng per embryo; ATG‐MO,4 ng per embryo. Primers spanning stx12 exon2 and exon3

(forward primer:5′‐CACACTGAATACCGCTCAAATC‐3′) and exon5
(reverse primer:5′‐CCACTGACTCCTTCTCTTTCTC‐3′)

were used for RT‐PCR analysis for confirmation of the efficacy of the E4I4‐MO. The primer ef1α sequences used as the internal control were 5′‐GGAAATTCGAGACCAGCAAATAC‐3′(forward) and 5′‐GATACCAGCCTCAAACTCACC‐3′(reverse).

### Mice

All animal care and experiments were performed in accordance with the Institutional Animal Care and Use Committee of the first affiliated hospital of Zhengzhou University guidelines (2024‐KY‐0399‐001). *Stx12*‐KO mice were generated by Shanghai Model Organisms Center, Inc. (Shanghai, China) as previously described.^[^ ^19^ ^]^ *Stx12^flox^* and *CTnT‐Cre* mice were generated by Shanghai Model Organisms Center, Inc. (Shanghai, China). Cardiac‐specific *Stx12* knockout mice were generated by mating *CTnT‐Cre* mice with *Stx12^flox^* mice. All mice were maintained in a 12‐h light‐dark cycle at 22 °C. All experiments were conducted using male mice during the light cycle.

### Echocardiography Analysis

Mice aged 3 months were anesthetized with isoflurane and placed on a heating pad to maintain body temperature. Echocardiography was conducted using echocardiography (Vevo2100 Imaging System), and cardiac function was assessed by detecting the ejection fraction (EF%) and fractional shorting (FS%) based on M‐mode recordings.

### Primary Cardiomyocyte Culture

On postnatal day 0 (P0), the hearts of C57BL mice were dissected and cut into small pieces. These pieces were then washed using HBSS. In order to disperse the tissues, trypsin (Sigma‐Aldrich) was applied for 5 min, followed by collagenase II (Sigma‐Aldrich) for an additional 30 min. The cells were further dissociated using fire‐polished pipettes before being plated onto 12 mm coverslips (Glasswarenfabrik Karl Hecht, Germany) that were coated with matrix gel (Corning). The plated cardiomyocytes were cultured in a plating medium and received feeding twice a week. Within 24 h, the cardiomyocytes began to exhibit autonomous beating. Electrophysiology recordings were conducted on cardiomyocytes at DIV3‐4. For AAV transduction, the virus was introduced to the medium at DIV2‐3 and observed under imaging at DIV7‐8.

### Cardiomyocyte Electrophysiology

At room temperature, a customized opto‐electro system was utilized for whole‐cell patch clamp recordings. The system consisted of an Axopatch 700B amplifier (Molecular Devices), a Digidata 1440A digitizer (Molecular Devices), a monochromator (Optoscan, Cairn Research Ltd., UK), and an imaging system (Olympus). Device control was managed by a customized Micro‐Manager software. Data were collected at a sampling rate of 10 kHz. Micropipettes were created by pulling filamented glass capillaries (Sutter Instrument, BF150‐86‐10) using a micropipette puller (Sutter Instrument, P1000), resulting in a tip resistance of 5–8 MΩ. The micropipette was filled with intracellular buffer (comprising 120 mm potassium gluconate, 3 mm KCl, 10 mm HEPES, 8 mm NaCl, 0.5 mm CaCl_2_, 5 mm EGTA, 2 mm ATP‐Mg, 0.3 mm GTP, pH 7.2) and positioned using a micromanipulator (Sutter, MP285).

### Western Blot Analysis

For the verification of *Stx12*‐KO mice, whole mouse tissue was homogenized in cold lysis buffer (1% Triton X‐100 and 1% DOC in Tris‐buffered saline buffer) containing phosphatase and protease inhibitors. This homogenization step was carried out using microtissue grinders (Kimble 749540‐0000, USA). The homogenates were then incubated on ice for 30 min to ensure complete cell lysis. Subsequently, the samples were centrifuged at 12 000 rpm for 15 min at 4 °C.

For *Stx12*‐cKO mice, proteins were obtained from isolated heart tissues using RIPA lysis buffer (Beyotime) supplemented with Protease Inhibitor Cocktail (Roche) and Phosphatase Inhibitor Cocktail (Roche). Protein concentration was determined using a BCA protein assay kit (Thermo Fisher) following the manufacturer's instructions.

A total of 20 µg of protein was subjected to SDS‐PAGE under reducing conditions. The separated proteins were then transferred onto a Polyvinylidene difluoride (PVDF) membrane (Millipore). After blocking the membrane with 5% milk for 2 h, it was incubated overnight at 4 °C with the appropriate primary antibody. Following primary antibody incubation, the membrane was incubated with a secondary antibody at room temperature for 2 h. The immunoblots were visualized using ECL Western Blotting Substrate (Pierce, Cat No: 32109) and exposed to an imager (GE, ImageQuant LAS 4000 mini).

The antibodies used in this study included syntaxin12 (Abcam, Cat No:ab13261 or customized by Proteintech), β‐Tubulin (Sigma, Cat No: T4026), GFP (Abmart, Cat No: P30010), β‐MHC (Abclonal, A7465), α‐MHC (Abclonal, A12964), phos‐S6K (CST, 9205), S6K (CST, 9202), oxphos (Abcam, ab110413), LC3b (CST, 2775), phospholamban (CST, 14562), p‐phospholamban (CST, 8496), PKA C‐α (CST, 5842), Phospho‐PKA C‐α (CST, 5661), Phospho‐CaMKII (CST, 12716), CaMKII delta (Proteintech, 20667‐1‐AP), TFEB (CST, 4240), p‐TFEB (CST, 37681), PGC1α (Proteintech, 66369‐1‐Ig), SERCA2 (thermo, MA3‐910), α‐Tubulin (Abcam, ab4074), GAPDH (Proteintech, 10494‐1‐AP or Sigma, G8795). Lamin A+Lamin C (Abcam, ab169532), Mitofilin (Proteintech, 10179‐1‐AP), GM130 (BD Biosciences, 610822), Calreticulin (Abcam, ab92516), Tom20 (Abcam, ab186735), OPA1 (BD Biosciences, 612607), and Hsp60 (Enzo Life Sciences, ADI‐SPA‐806).

### Real‐Time Quantitative PCR

RNA was extracted from heart tissues or collected cells using Trizol Reagent (Invitrogen) according to the manufacturer's instructions. After concentration detection, 1 µg of total RNA was reversely transcribed to cDNAs using PrimeScript RT reagent Kit (TaKaRa) following the manufacturer's instructions. Then cDNAs were amplified using a SYBR‐Green Quantitative PCR kit (Takara) with an iCycler IQ system (Biorad). GAPDH was used as endogenous control. The value relative to the control sample is shown by 2‐ΔΔCT. The sequences of primers used for qRT‐PCR were listed as follows:

**Table:** 

Gene	Forward primer	Reverse primer
Nppb	GAGGTCACTCCTATCCTCTGG	GCCATTTCCTCCGACTTTTCTC
β‐MHC	TGTCCAGCAGGTGTCATACG	TTGCATTGATGCGTGTCACC
Skeletal α‐actinin	ATCGCTGACCGCATGCAGAA	TGCGCCTAGAAGCATTTGCGGT
GAPDH	ACTCCACTCACGGCAAATTC	TCTCCATGGTGGTGAAGACA

### ECG Measurement

Mice aged 3 months were anesthetized before recording. ECG was recorded by a 4‐lead ECG system (iWorx, BIO4). Electrodes were plugged into the four limbs of the mouse according to the manufacturer's manual.

### HE and WGA Staining

Tissues were harvested and rinsed in 0.01 m PBS, followed by fixation in 4% PFA for 12 h. This preparation was done to obtain either paraffin sections or frozen sections. Paraffin‐embedded sections of heart cryosections were stained with H&E using the standard process.

For wheat germ agglutinin (WGA) staining, cross cryosections of the heart were incubated with WGA conjugated to Alexa Fluor 594 (Invitrogen, W21405) overnight at 4 °C. The sections were then washed three times for 15 min each with 0.01 m PBS. Subsequently, the sections were stained with DAPI (Invitrogen) and mounted with fluoromount‐G (SouthernBiotech, 0100‐01) for imaging.

To measure the cross‐sectional area of myocytes in the right and left ventricles, the WGAv3‐1 plugin of ImageJ was utilized. At least 100 cardiomyocytes were measured per image, and analysis was performed on over 5 sections obtained from at least 4 to 6 hearts per genotype.

### Mitochondrial Cristae Density Analysis

Mitochondrial cristae density analysis was performed using ImageJ. A straight line was drawn to connect the furthest endpoint within each mitochondrion, and the number of junctions between the straight line and mitochondrial cristae was counted. The relative quantification of mitochondrial cristae density was determined by dividing the number of junctions by the distance of the straight line.

### Electron Microscopy

The tissues were removed and quickly rinsed in cold 0.1 m PBS. They were then fixed in a 2.5% glutaraldehyde buffer at 4 °C for 1.5 h. Afterward, the tissues were washed three times for 10 min each with 0.01 m PBS and post‐fixed for 1 h in a 2% osmium tetroxide solution. Following another wash with 0.01 m PBS, the tissues were dehydrated through a gradient series of ethanol. Next, the tissues were pre‐infiltrated with a mixture of acetone and epon at room temperature for 1.5 h, followed by infiltration with pure epon. They were then embedded and polymerized overnight in a 37 °C oven, followed by 12 h in a 45 °C oven, and finally, 48 h in a 60 °C oven. The epon blocks were trimmed, and ultrathin sections (70 nm) were cut using a Leica EM UC7. These sections were counterstained with uranyl acetate and lead citrate and examined using a transmission electron microscope (FEI Tencnai G2 Spirit Twin).

### Metal Content Measurements

The total metal content of the heart was analyzed using inductively coupled plasma mass spectrometry (ICP‐MS). The heart tissues of mice were weighed and then digested with nitric acid in a microwave (CEM, Mars). The resulting solution was diluted with 2% nitric acid, and the excess acid was evaporated. The remaining solution was further diluted to 2 mL with 2% nitric acid for analysis by ICP‐MS with MS detection (Agilent 7700X series). The total metal content was normalized by dividing it by the wet weight of the tissues.

### ATP Assay

The hearts of P0 C57BL mice were isolated and weighted. Then the hearts were cut into small pieces and homogenated on ice in lysate buffer provided by ATP Assay Kit (Beyotime, S0027) using a Dounce homogenizer. ATP content was determined according to the instructions of manufacturers. The experiments were performed more than 3 times, and the results were presented as mean ± SEM.

### Subcellular Fractionation

Subcellular fractions were also obtained from the mouse heart. The nucleus and cytosol were extracted by Cytoplasmic Protein Extraction Kit (Beyotime, P0027). Golgi apparatus was extracted by Minute Golgi Apparatus Enrichment Kit (Invent, GO‐037). The endoplasmic reticulum was extracted by the Endoplasmic Reticulum Isolation Kit (Sigma, ER0100). Mitochondria were extracted using an extraction buffer containing 250 mm sucrose, 5 mm HEPES, and 1 mm EGTA at pH 7.4. The mouse heart was cut into small pieces and manually homogenized at 4 °C using a glass homogenizer with the extraction buffer. The homogenization was performed 20 times, ensuring to avoid excessive homogenization to prevent damage to the mitochondrial membrane. After homogenization, the solution was transferred to a 1.5 mL EP tube and centrifuged at 3000 rpm at 4 °C for 5 min. The supernatant was carefully collected, and any unbroken cells and nuclei were discarded. The supernatant was then subjected to a second centrifugation at 3000 rpm for 5 min at 4 °C to remove any remaining precipitates until no further precipitation was observed at the bottom of the EP tube. The supernatant was further centrifuged at 13 000 rpm for 30 min at 4 °C to obtain crude mitochondria. These mitochondria were washed twice with mitochondria extraction buffer and centrifuged at 13 000 rpm for 5 min each time to obtain relatively pure mitochondria. The subcellular fractions were analyzed by Western blotting.

### Proteinase K Digestion Assay

Proteinase K digestion was carried out by suspending isolated mitochondria in an extraction buffer and incubating them with 5 µg mL^−1^ proteinase K at 37 °C for 15 min. The digestion was stopped by adding 2 mm phenylmethylsulfonyl fluoride (PMSF). The mitochondrial proteins were analyzed by Western blotting.

### Calcium Imaging

Cardiomyocytes were loaded with Fluo‐4‐AM (Invitrogen, F14201) at a final concentration of 5 µm in Tyrode's buffer, followed by a 15‐min incubation at 37 °C. The fluorescence of Fluo4‐stained cells was observed using a fluorescence microscope (Olympus IX83, Japan) in Tyrode's buffer. Fluo4 was excited at 488 nm, and emission was collected at 490–540 nm.

### Virus Preparation and Transduction

To transfect cultured cardiomyocytes, the AAV DJ serotype virus was utilized. The production of this virus involved co‐transfecting gene plasmid, capsid (pAAV‐DJ), and helper plasmids (pHelper) into 293t cells using the calcium phosphate precipitation method. After an incubation period of 48 h, the viruses were collected from cell pellets using four cycles of frozen‐thaw techniques. The titer of the virus was determined by conducting real‐time PCR analysis.

### Voltage Imaging

Cardiomyocytes transfected with the cTnT‐SomArchon AAV virus were observed under a fluorescence microscope (Olympus IX83, Japan) in Tyrode's buffer. SomArchon was excited with a 633 nm laser, and emission was collected using a band filter of 650–720 nm.

### Drug Treatment

To block SERCA2 ATPase's activity, thapsigargin (Sigma, T9033) at a concentration of 100 nm was added to Tyrode's solution before calcium imaging experiments or electrophysiology recordings. For TMRE treatment, TMRE at 50 nm was added to the culture medium of cardiomyocytes, and the cells were incubated for 16 h before calcium imaging or electrophysiology recording. To treat with rapamycin, rapamycin (MCE, HY‐10219) at 50 nm was added to the culture medium of cardiomyocytes, and the cells were incubated for 16 h before calcium imaging or electrophysiology recording.

### Action Potential and Calcium Modeling

The Rasmusson model^[^ ^31^ ^]^ was employed to simulate the action potential and calcium dynamics of cardiomyocytes. The equations were written in ode form and could be simulated using the XPPAUT program. To investigate the effect of the SERCA ATPase pump rate on calcium the SERCA ATPase pump rate (ν3) was adjusted from 1 to 0.1 µm. The curve from the peak of the calcium to the 20% amplitude value was fitted using a one‐phase exponential decay equation (Equation (1)).

(1) $$y=A*e^{-\frac{x}{\tau }}$$

All the ode equations and parameters are listed in the Supporting Information.

### Rapamycin Intervention Experiments

Rapamycin was initially dissolved in DMSO to achieve a concentration of 20 mg mL^−1^. It was then diluted in 30% PEG300, 5% Tween 80, and saline, resulting in a final concentration of 0.5 mg mL^−1^, and subsequently sterile‐filtered. For rapamycin administration, mice aged 2–3 months received daily intraperitoneal injections of 5 µL g^−1^ body weight (2.5 mg kg^−1^) for one week. The control group was administered the vehicle.

### CDN1163 Treatment

CDN1163 (HY‐101455, MCE) was initially dissolved in DMSO to achieve a concentration of 100 mg mL^−1^. It was then diluted in corn oil (HY‐Y1888, MCE) to reach a final concentration of 10 mg mL^−1^. For CDN1163 administration, mice aged 3 months received daily intraperitoneal injections of 5 µL g^−1^ body weight (50 mg kg^−1^) for 5 days. Echocardiographic assessments were conducted both before and after CDN1163 treatment.

### SERCA ELISA Assay

After treatment with or without rapamycin or vehicle, hearts were isolated from anesthetized mice. After rinse with PBS, the hearts were weighed and cut into small pieces. The tissues were then subjected to a Dounce homogenizer for homogenization. The homogenate was further treated with an ultrasonic cell disruptor (Jingxin Industrial Development Co., Ltd, Shanghai) to break down the cells. The homogenate was centrifuged at 12 000 rpm, 4 °C, and the supernatant was collected. The supernatant was used for the ELISA assay. The SERCA level was measured by a Mouse SERCA ELISA assay kit (CoiBO Biotechnology, Shanghai) according to the manufacturer's instructions.

### Data and Statistical Analysis

All electrophysiology data were analyzed using pClampfit (Molecular Devices) in combination with customized Perl scripts. For calcium and voltage imaging analysis, the acquired images were analyzed with ImageJ, and the fluorescence curve was analyzed by customized Perl scripts to calculate the frequency, amplitude, and decay constant. For demonstrating RNA‐seq data, the volcano plot, and GO‐enrichment analysis were generated with customized Python scripts. All data in the main text were presented as mean ± SD. All curves were presented as mean ± SEM except boxplots. GraphPad was used for statistical analysis, with unpaired two‐tailed Student's *t‐test* employed for comparison of two samples.

## Conflict of Interest

The authors declare no conflict of interest.

## Author Contributions

R.‐Z.Y., F.L., and J.L. contributed equally to this work. Conceptualization, J.S.K.; Software: R.Z.Y.; Formal analysis: R.Z.Y., J.S.K.; Methodology: R.Z.Y., F.L., J.L., S.A.L., Z.B.W., D.H.L., J.S.K.; Investigation: R.Z.Y., F.L., J.L., S.A.L., Z.B.W., D.H.L.; Visualization: R.Z.Y., F.L., J.L., S.A.L., Z.B.W., J.S.K.; Funding acquisition: J.S.K., S.A.L., P.P.L.; Project administration: J.S.K.; Supervision: J.S.K., Y.Y.; Writing–original draft: R.Z.Y., F.L., J.L., S.A.L., J.S.K.; Writing–review and editing: R.Z.Y., B.Z., J.S.K.

## Supporting information



Supporting Information

## Data Availability

The data that support the findings of this study are available from the corresponding author upon reasonable request.
