# Supplementary material for: Rapamycin Alleviates Heart Failure Caused by Mitochondrial Dysfunction and SERCA Hypoactivity in Syntaxin 12/13 Deficient Models
Source: Adv Sci (Weinh). 2025 Jun 26;12(31):e07210. doi: 10.1002/advs.202507210 (PMC12376582; doi:10.1002/advs.202507210)
Supplement: Supplementary file 1 — Supporting Information [file ADVS-12-e07210-s001.pdf]

ADVANCED SCIENCE
Open Access

Supporting Information

for *Adv. Sci.*, DOI 10.1002/advs.202507210

Rapamycin Alleviates Heart Failure Caused by Mitochondrial Dysfunction and SERCA Hypoactivity in *Syntaxin 12/13* Deficient Models

Run-Zhou Yang, Fang Li, Jiao Liu, Shu-Ang Li, Dan-Hua Liu, Zhuanbin Wu, Pei-Pei Liu, Wenju Liu, Bin Zhou, Cizhong Jiang, Haibing Zhang, Ying Yu and Jian-Sheng Kang**

Rapamycin alleviates heart failure caused by mitochondrial dysfunction and SERCA hypoactivity in *Syntaxin 12/13* deficient models

Run-Zhou Yang[1,9], Fang Li[2,9], Jiao Liu[3,4,9], Shu-Ang Li[1], Dan-Hua Liu[1], Zhuanbin Wu[5], Pei-Pei Liu[1], Wenju Liu[7], Zhou bin[6], Cizhong Jiang[7], Haibing Zhang[8], Ying Yu[3*], Jian-Sheng Kang[1]*

Affiliations:

[1] Clinical Systems Biology Laboratories, The First Affiliated Hospital of Zhengzhou University; Zhengzhou 450052, China.

[2] Department of Anesthesiology, Shanghai General Hospital, Shanghai Jiao Tong University School of Medicine; Shanghai 200000, China

[3] Department of Pharmacology and Tianjin Key Laboratory of Inflammation Biology, School of Basic Medical Sciences, Tianjin Medical University; Tianjin 300070, China.

[4] Department of Cardiology, First Affiliated Hospital of Zhengzhou University; Zhengzhou, 450052, China.

[5] Shanghai Model Organisms Center, Inc., Shanghai, China.

[6] Shanghai Institute of Biochemistry and Cell Biology, Center for Excellence in Molecular Cell Science, Chinese Academy of Sciences, Shanghai, China

[7] School of Life Sciences and Technology, Tongji University, Shanghai, China.

[8] Shanghai Institute of Nutrition and Health, Chinese Academy of Sciences, Shanghai, 200031 China

[9] These authors contributed equally.

* Correspondences should be addressed to kjs@zzu.edu.cn (J.S.K.) and yuying@tmu.edu.cn (Y.Y.).

Jian-Sheng Kang, Clinical Systems Biology Laboratories, The First Affiliated Hospital of Zhengzhou University, Zhengzhou, 450052, China. ORCID ID: 0000-0002-2603-9718.

Ying Yu, Department of Pharmacology, Tianjin Key Laboratory of Inflammatory Biology, Center for Cardiovascular Diseases, Haihe Laboratory of Cell Ecosystem, Key Laboratory of Immune Microenvironment and Disease (Ministry of Education), The Province and Ministry Co-sponsored Collaborative Innovation Center for Medical Epigenetics, School of Basic Medical Sciences, Tianjin Medical University, Tianjin, China. ORCID ID: 0000-0002-6476-1752

Supplementary Figures and Tables:

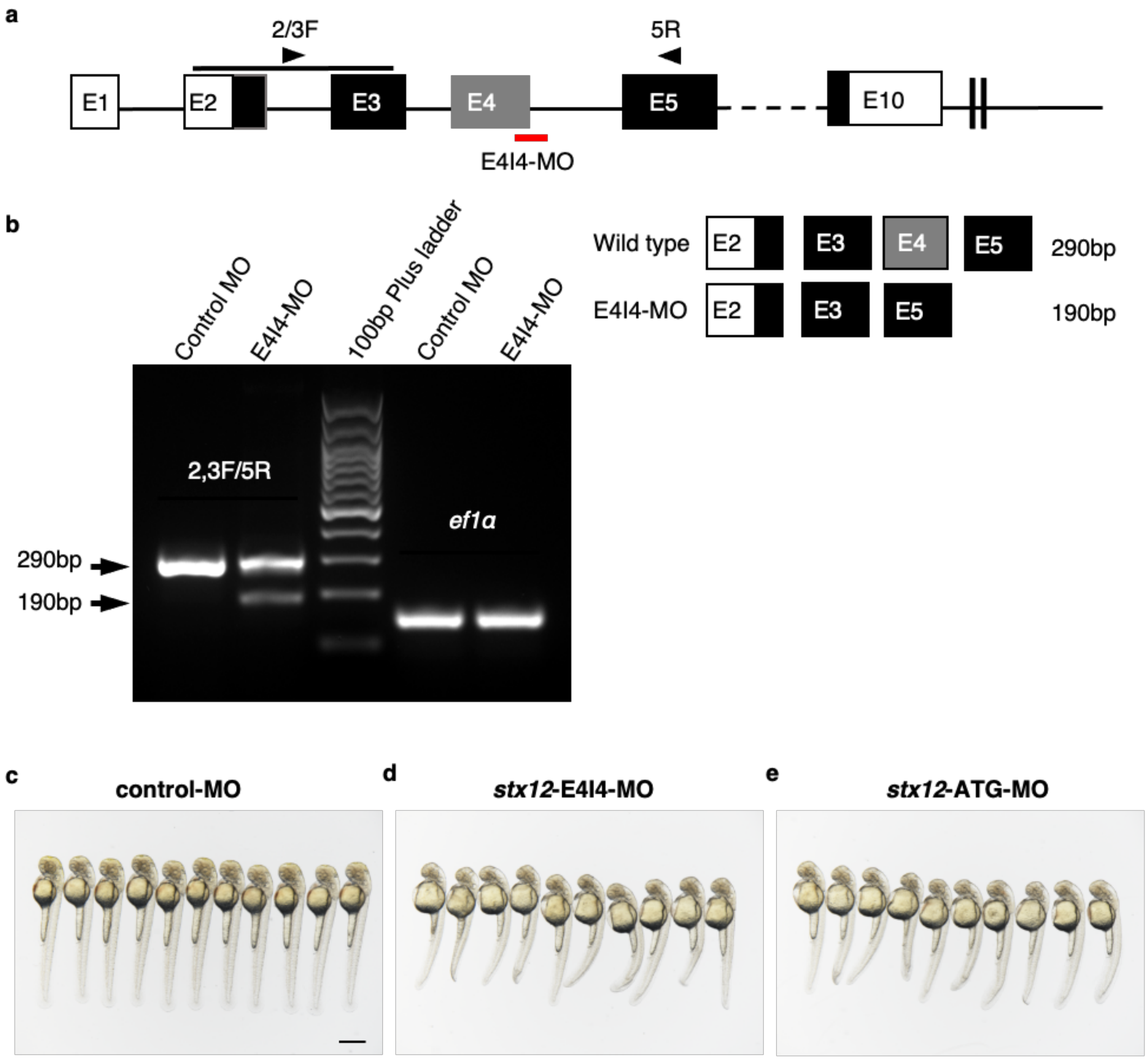

Supplementary Figure S1. Information about *Stx12* knockdown in zebrafish.

(a) The zebrafish *Stx12* gene was targeted by specific morpholino antisense to prevent proper splicing of exon 4 (E4I4-MO). Primers 2/3 F and 5R interrogate the presence of wild-type (non-mutant) transcripts or those in which exon 4 has been skipped. **(b)** RT-PCR of *Stx12* transcript from control and E4I4-MO morpholino-injected embryos 2 days after fertilization, demonstrating skipping of exon 4. Injection of 4 ng of *Stx12* morpholino alters the splicing between exon 4 and intron 4, as revealed by a shift in PCR bands between control (290 bp) and *Stx12* morpholino injected embryos (190 bp). **(c)** Morphology of representative zebrafish larvae at 2 days post-fertilization (dpf) following controlled morpholino injection. Scale bar, 500 μm. **(d)** Morphology of representative zebrafish larvae at 2 days post-fertilization (dpf) following stx12-E4I4 morpholino injection. **(e)** Morphology of representative zebrafish larvae at 2 days post-fertilization (dpf) following stx12-ATG morpholino injection.

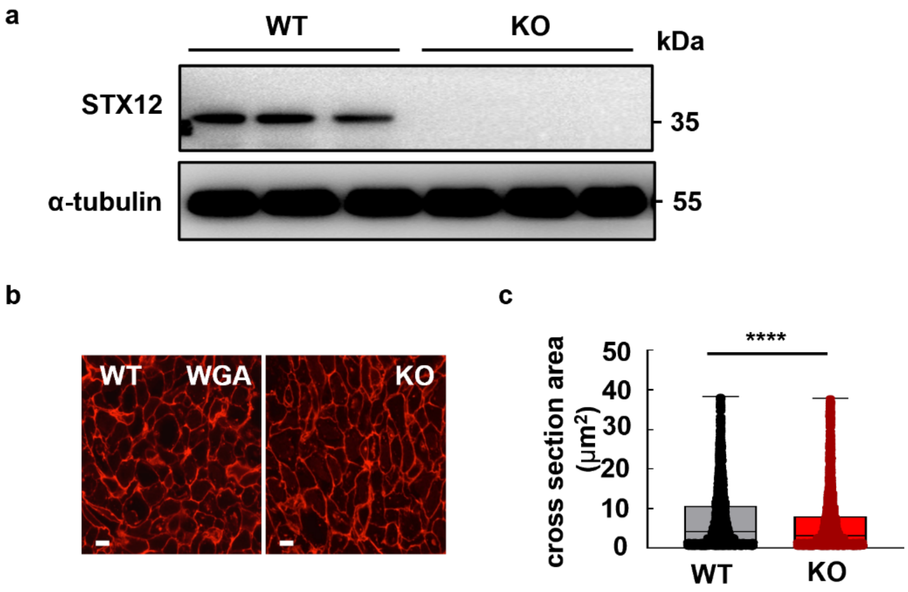

Supplementary Figure S2. Verification of STX12 expression in *Stx12*-KO mouse and WGA staining. (a) Immunoblot analysis of cardiac STX12 expression of wildtypes (WT) and homozygotes (KO) mice. Cardiac protein samples prepared from P0 littermates are blotted with antibodies against STX12 (top) and loading control α-tubulin (bottom). **(b)** WGA staining of cardiac cross section. Representative *Stx12-KO* WGA staining on the right, and wild-type heart (WT) on the left. The scale bars represent 5 μm. **(c)** Quantification of cross-section area in wildtypes (WT) and *stx12*-knockout (KO) mice (WT, number of mice = 6, WGA staining area = 6.96 ± 7.29 μm^2 (mean ± s.d), number of WGA area = 3176; KO, number of mice = 4, WGA staining area = 5.87 ± 6.87 (mean ± s.d.), number of WGA area = 2734; *t-test*, $p < 0.0001$).

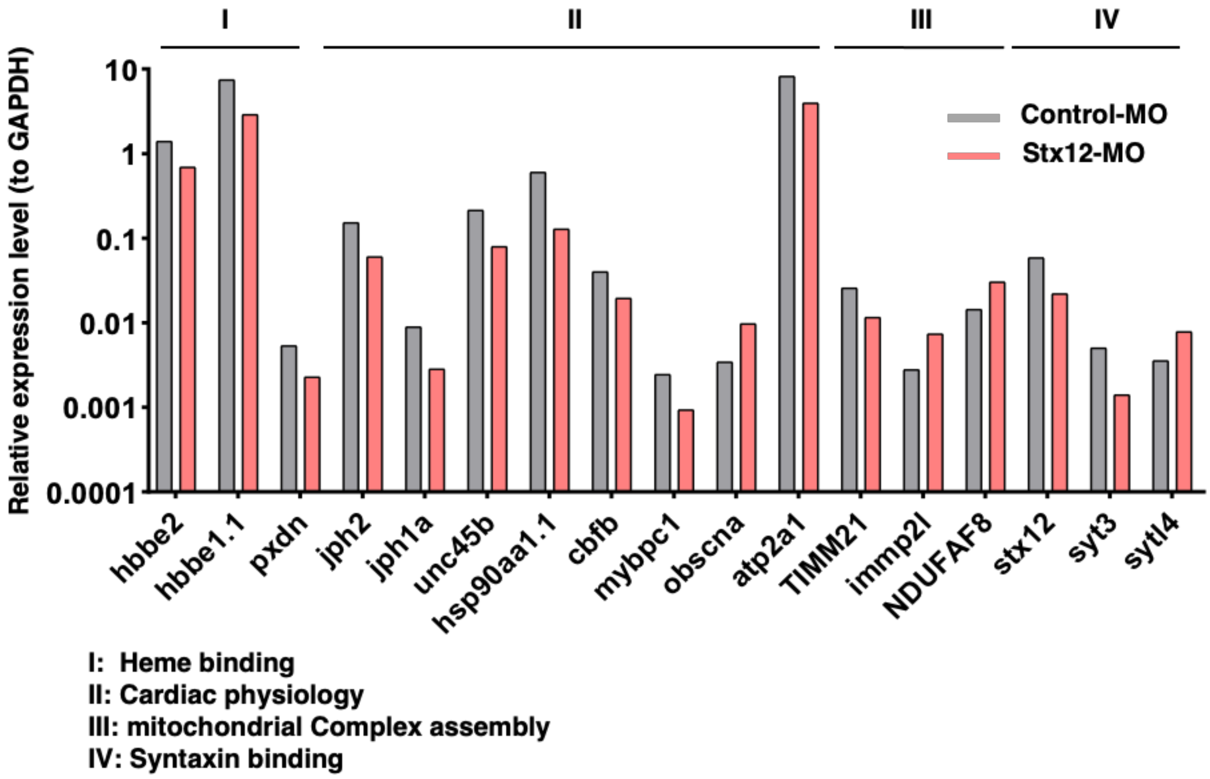

Supplementary Figure S3. ***Stx12*** **deficiency affected genes that are involved in various pathways.** Zebrafish RNAseq data demonstrated that genes that participate in heme binding (I), cardiac physiology (II), mitochondrial complex assembly (III), and syntaxin binding (IV) were significantly affected. The $\log_2$fold change and $-\log_{10}$p-value of each genes were as follows: hbbe2, -1.02, 51.83; hbbe1.1, -1.37, 101.10; jph2, -1.34, 75.97; jph1a, -1.53, 20.70; unc45b, -1.43, 94.74; hsp90aa1.1, -2.21, 225.03; cbfb, -1.03, 33.33; mybpc1, -1.09, 6.44; atp2a1, -0.50, 17.60; TIMM21, -1.12, 12.36; immp2l, 1.28, 9.76; NDUFAF8, 1.06, 5.51; stx12, -1.40, 44.75; syt3, -1.58, 13.71; sytl4, 1.06, 8.20.

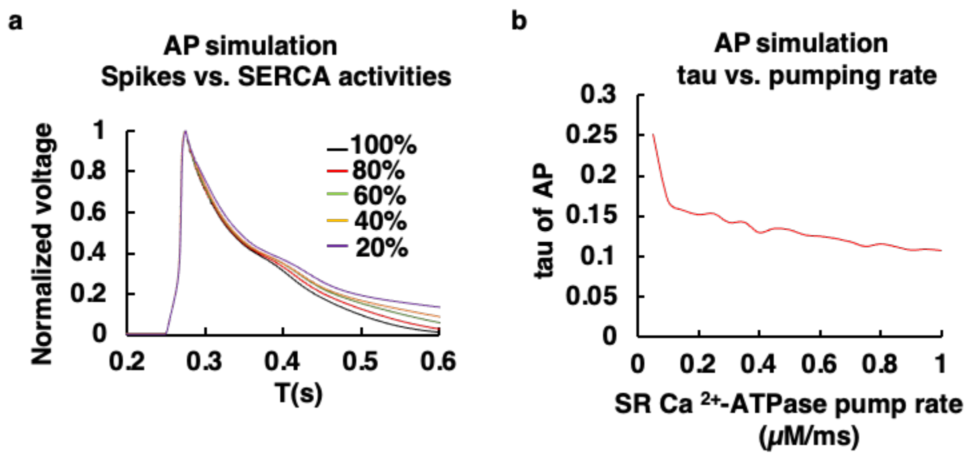

Supplementary Figure S4. Computational modeling of cardiomyocytes firing under SERCA ATPase deficiency. (a) The voltage curves under different SERCA pump activities were obtained through computer model simulation. **(b) The c**urve of tau constants of the decay phase of action potential curves obtained through single exponential fitting under different SERCA pumping activities.

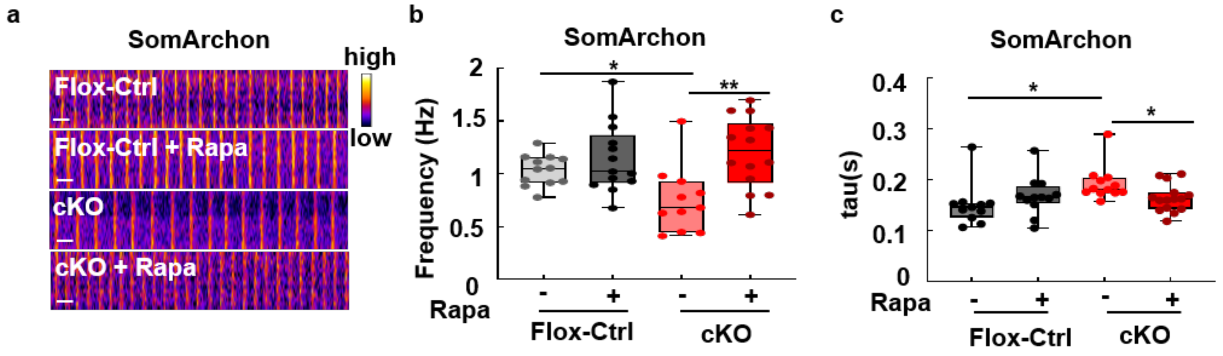

Supplementary Figure S5. Action potential imaging demonstrated the protective effect of rapamycin in *Stx12*-deficient cardiomyocytes. (a) Kymograph of the membrane voltage changes in cultured *stx12*-cKO cardiomyocytes with or without rapamycin treatment. cTnT-SomArchon was expressed in cardiomyocytes for monitoring voltage dynamics. Images were presented as pseudo colors. Time scale bar, 1 s. **(b)** Quantification of action potential frequency obtained through voltage imaging (control + vehicle, n=12; control + rapamycin, n=13; cKO + vehicle, n= 11; cKO + rapamycin, n=14). The beating frequency of *stx12*-ckO is significantly decreased compared to the wildtype (*t-test*, p = 0.0102, *). After rapamycin administration, *stx12*-cKO showed a significant increase in beating frequency (*t-test*, p = 0.0022, **). **(c)** Comparisons of decay constant (τ) obtained by single exponential fitting the declining portion of the voltage change curve. *Stx12*-ckO showed a significant increase in tau compared to the control (*Stx12*-flox) (p =0.0131, *). After adding rapamycin, cKO showed a significant decrease in tau (p = 0.0213, *).

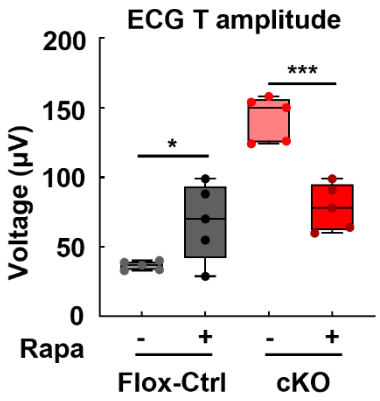

Supplementary Figure S6. Rapamycin intervention improved heart failure in *Stx12*-cKO mice. Changes in T-wave of electrocardiogram (ECG) in control (*Stx12*-flox) and *Stx12*-CKO mice before and after rapamycin treatment (Flox-control + vehicle, n=5; Flox-control + rapamycin, n= 5; cKO + vehicle, n=5; cKO + rapamycin, n=5; WT vehicle vs. cKO vehicle, *t-test*, $p = 0.0347$, *; cKO + vehicle vs. cKO + rapamycin, *t-test*, $p = 0.0003$, ***).

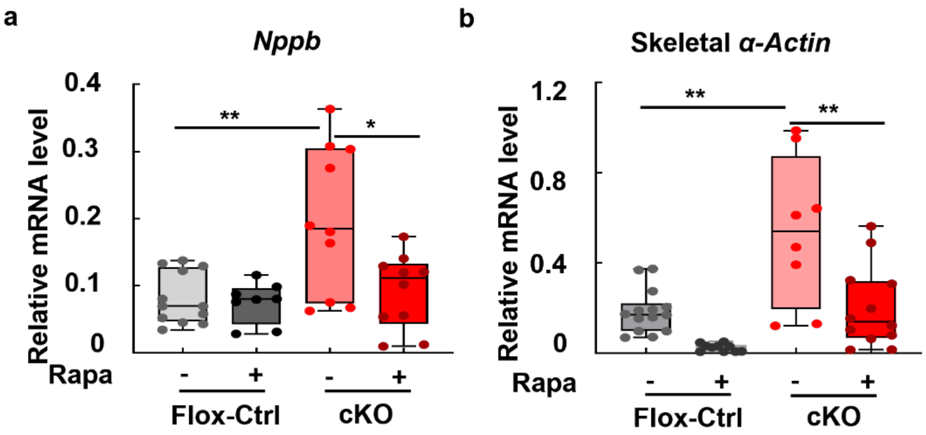

Supplementary Figure S7. Rapamycin intervention suppressed the markers of cardiac hypertrophy in *Stx12*-cKO mice. **(a)** qRT-PCR analysis of mRNA changes of Nppb in the heart after rapamycin or vehicle treatment (Flox-control + vehicle, n = 14; Flox-control + rapamycin, n = 10; cKO + vehicle, n = 10; cKO + rapamycin, n = 12; control + vehicle vs. cKO + vehicle, $p = 0.0024$, **; cKO + vehicle vs. cKO + rapamycin, p = 0.0131, *). **(b)** qRT-PCR analysis of mRNA changes of skeletal α-Actin in the heart after rapamycin or vehicle treatment (Flox-control + vehicle, n = 14; Flox-control + rapamycin, n = 12; cKO + vehicle, n = 12; cKO + rapamycin, n = 10; Flox-control + vehicle vs. cKO + vehicle, $p = 0.0175$, *; cKO + vehicle vs. cKO + rapamycin, $p = 0.0248$, *).

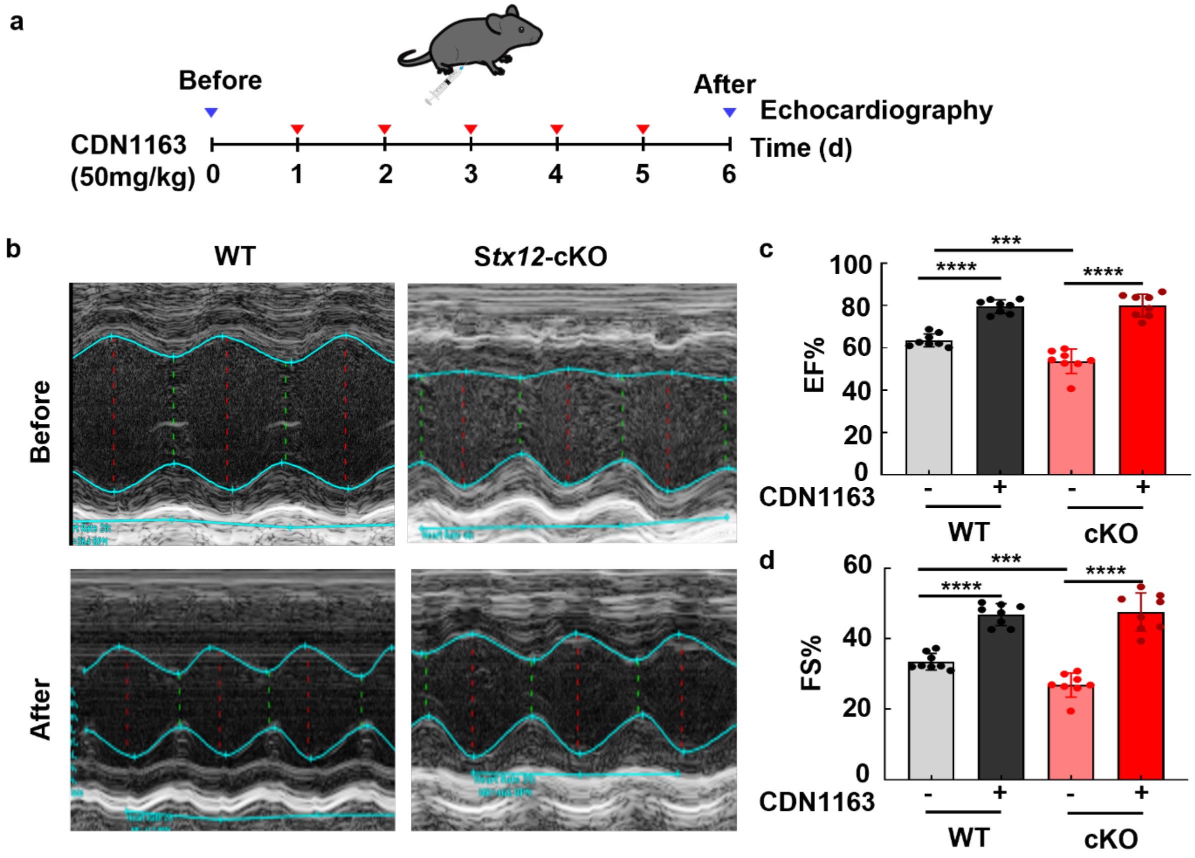

Supplementary Figure S8. CDN1163 treatment enhanced cardiac function in *Stx12*-cKO mice. (a) Schematic diagram of CDN1163 injection in mice. Cardiac functions were evaluated by echocardiography before and after intraperitoneally daily injection in *Stx12*-cKO (*Stx12*$^{flox/flox}$ with *CTnT-Cre*) and wild-type mice for 5 days. **(b)** Representative M-mode echocardiography of left ventricular chamber of wildtype (WT) and *Stx12*-cKO mice before and after CDN1163 treatment. **(c, d)** Changes in ejection fraction (EF%) (c) and fractional shortening (FS%) (d) of the left ventricle in wildtype (WT) and *Stx12*-cKO mice before and after CDN1163 treatment. EF% was significantly increased in WT and *Stx12*-cKO mice after CDN1163 treatment (WT, n = 8; WT + CDN1163, n = 8, *t-test*, $p < 0.0001$; cKO, n = 8, cKO + CDN1163, n = 8, *t-test*, $p < 0.0001$). FS% was significantly increased in WT and *Stx12*-cKO mice after CDN1163 treatment (WT, n = 8; WT + CDN1163, n = 8; cKO, n = 8; cKO + CDN1163, n = 8; WT and WT + CDN1163, $p < 0.0001$; cKO and cKO + CDN1163, $p < 0.0001$; WT and cKO, $p = 0.0005$). Statistical results: ***p < 0.001, ****p < 0.0001; *t-test*.

Supplementary Table S1 Designing of Zebrafish *Stx12* Morpholino. The zebrafish *Stx12* gene was targeted by two specific morpholino antisense strategies to prevent either the translation of the zebrafish gene (ATG-MO) or proper splicing of exon4 (E4I4-MO).

Morpholino Name	Morpholino oligo sequence
stx12-E4I4-MO (E4I4-MO)	5'-ACTGGCAACTACAAAAGTACCTGTT-3'
stx12-ATG-MO (ATG-MO)	5'-TGGAGCAAACTACAGCAGGAAGCCA-3'
Standard Control MO	5'- CCTCTTACCTCAGTTACAATTTATA-3'

Supplementary Table S2 Microelement contents (iron, copper, and zinc) in heart of wildtypes (WT) and *Stx12*-KO homozygous mice (Homo). Data are represented in mean ± SD. * represents a significant difference ($P < 0.05$).

Elements (μg/g)	Genotype		***P* value, *t* test**
	**WT (+/+)**	**Homo (-/-)**	**WT vs. Homo**
**Iron**	255.97 ± 66.4 (n=12)	178.91 ± 52.02 (n=11)	0.0057*
**Copper**	3.65 ± 0.53 (n=12)	3.95 ± 0.66 (n=12)	0.2456
**Zinc**	14.72 ± 1.98 (n=12)	10.89 ± 3.07 (n=11)	0.0017*

Supplementary Table S3. The decay constants of calcium signals and calculated SERCA activity in primary cultured cardiomyocytes. SERCA activity was calculated based on the relationship between tau and pumping rate (Figure 5i and S6B).

Cardiomyocyte	Ca^{2+}-tau (s)	SERCA2 activity (μM/ms)
WT	0.16±0.0087	0.8841
Stx12-KO	0.28±0.0086	0.4060
WT, rapamycin (50 nM)	0.22±0.021	0.5927
Stx12-KO, rapamycin (50 nM)	0.19±0.0098	0.7186
WT, TG (100 nM)	0.53±0.0703	-0.0222
WT, TMRE (50 nM)	0.37±0.0467	0.2030

Supplementary Text

The following equations (1-115) are used to build the model for cardiomyocytes. The model is based on a set of 41 ordinary differential equations (ode)[30] . The definitions of parameters in equations are provided in supplementary table 4. ν_3 (in equation 87) is changed from 0-1 in the stimulation.

$$E_K = \frac{RT}{F}\ln\frac{[K_o]}{[K_i]} \tag{1}$$

$$C_m\frac{dV}{dt} = -I_{CaL} + I_{pCa} + I_{NaCa} + I_{Cab} + I_{Na} + I_{Nab} + I_{NaK} + I_{Kto,f} + I_{Kto,s} + I_{K1} + I_{Ks} + I_{Kur} + I_{Kss} + I_{Kr} + I_{Cl,Ca} - r_{stat} \tag{2}$$

$$\frac{d[K_i]}{dt} = -I_{Kto,f} + I_{Kto,s} + I_{K1} + I_{Ks} + I_{Kss} + I_{Kur} + I_{Kr} - 2I_{NaK}\frac{A_{cap}C_m}{V_{myo}F} \tag{3}$$

$$I_{Kto,f} = G_{Kto,f}a_{to,f}^3 i_{to,f}(V - E_K) \tag{4}$$

$$\alpha_a = 0.18064\,e^{0.03577(V+30.0)} \tag{5}$$

$$\beta_a = 0.3956\,e^{-0.06237(V+30.0)} \tag{6}$$

$$\alpha_{i1} = \frac{0.000152\,e^{-(V+13.5)/7}}{0.0067083\,e^{-(V+33.5)/7} + 1} \tag{7}$$

$$\beta_{i1} = \frac{0.00095\,e^{(V+33.5)/7}}{0.051335\,e^{(V+33.5)/7} + 1} \tag{8}$$

$$\frac{da_{to,f}}{dt} = \alpha_a(1 - a_{to,f}) - \beta_a a_{to,f} \tag{9}$$

$$\frac{di_{to,f}}{dt} = \alpha_{i1}(1 - i_{to,f}) - \beta_{i1} i_{to,f} \tag{10}$$

$$I_{Kto,s} = G_{Kto,s}a_{to,s}i_{to,s}(V - E_K) \tag{11}$$

$$\frac{da_{to,s}}{dt} = \frac{a_{ss} - a_{to,s}}{\tau_{ta,s}} \tag{12}$$

$$\frac{di_{to,s}}{dt} = \frac{i_{ss} - i_{to,s}}{\tau_{ti,s}} \tag{13}$$

$$a_{ss} = \frac{1}{e^{-(V+22.5)/7.7} + 1} \tag{14}$$

$$i_{ss} = \frac{1}{e^{-(V+45.2)/5.7} + 1} \quad (15)$$

$$\tau_{ta,s} = 2.058 + 0.493\, e^{-0.0629V} \quad (16)$$

$$\tau_{ti,s} = 270.0 + \frac{1050.0}{1 + e^{(V+45.2)/5.7}} \quad (17)$$

$$I_{K1} = 0.2938\left(\frac{[K_o^+]}{[K_o^+] + 210.0}\right)\left[\frac{V - E_K}{1 + e^{0.0896(V-E_K)}}\right] \quad (18)$$

$$I_{Ks} = G_{Ks} n_{Ks}^2 (V - E_K) \quad (19)$$

$$\frac{dn_{Ks}}{dt} = \alpha_n(1 - n_{Ks}) - \beta_n n_{Ks} \quad (20)$$

$$\alpha_n = 4.81333 \times 10^{-6}(26.5 + V)\left[1.0 - e^{-0.128(V+26.5)}\right] \quad (21)$$

$$\beta_n = 9.53333 \times 10^{-5} e^{-0.038(V+26.5)} \quad (22)$$

$$I_{Kur} = G_{Kur} a_{ur} i_{ur} (V - E_K) \quad (23)$$

$$\frac{da_{ur}}{dt} = \frac{a_{ss} - a_{ur}}{\tau_{aur}} \quad (24)$$

$$\frac{di_{ur}}{dt} = \frac{i_{ss} - i_{ur}}{\tau_{iur}} \quad (25)$$

$$\tau_{aur} = 2.058 + 0.493\, e^{-0.0629V} \quad (26)$$

$$\tau_{iur} = 1200.0 - \frac{170.0}{1.0 + e^{(V+45.2)/5.7}} \quad (27)$$

$$I_{Kss} = G_{Kss} a_{Kss} i_{Kss} (V - E_K) \quad (28)$$

$$\frac{da_{Kss}}{dt} = \frac{a_{ss} - a_{Kss}}{\tau_{Kss}} \quad (29)$$

$$\frac{di_{Kss}}{dt} = 0 \quad (30)$$

$$\tau_{Kss} = 13.17 + 39.3 e^{-0.0862V} \quad (31)$$

$$I_{Kr} = G_{Kr} O_K \left[V - \frac{RT}{F} \ln \left(\frac{0.98[K^+]_o + 0.02[Na^+]_o}{0.98[K^+]_i + 0.02[Na^+]_i} \right) \right] \tag{32}$$

$$C_{K0} = 1 - (C_{K1} + C_{K2} + O_K + I_K) \tag{33}$$

$$\frac{dC_{K1}}{dt} = -\left(\beta_{a0} C_{K1} + k_f C_{K1}\right) + \alpha_{a0} C_{K0} + k_b C_{K2} \tag{34}$$

$$\frac{dC_{K2}}{dt} = -(k_b C_{K2} + \alpha_{a1} C_{K2}) + \beta_{a1} O_K + k_f C_{K1} \tag{35}$$

$$\frac{dO_K}{dt} = -(\beta_{a1} O_K + \alpha_i O_K) + \alpha_{a1} C_{K2} + \beta_i I_K \tag{36}$$

$$\frac{dI_K}{dt} = \alpha_i O_K - \beta_i I_K \tag{37}$$

$$\alpha_{a0} = 0.022348 e^{0.01176V} \tag{38}$$

$$\alpha_{a1} = 0.013733 e^{0.038198V} \tag{39}$$

$$\beta_{a0} = 0.047002 e^{-0.0631V} \tag{40}$$

$$\beta_{a1} = 6.89 \times 10^{-5} e^{-0.04178V} \tag{41}$$

$$\alpha_i = 0.090821 e^{0.023391(V+5.0)} \tag{42}$$

$$\beta_i = 0.006497 e^{-0.03268(V+5.0)} \tag{43}$$

$$I_{NaK} = I_{NaK}^{max} f_{NaK} \left(\frac{[K]_o}{[K]_o + K_{m,Ko}} \right) \cdot \{ \frac{1}{1 + \left(K_{m,Nai}/[Na^+]_i\right)^{3/2}} \} \tag{44}$$

$$f_{NaK} = \frac{1}{1 + 0.1245\, e^{-0.1VF/RT} + 0.0365\, \sigma\, e^{-0.1VF/RT}} \tag{45}$$

$$\sigma = \frac{1}{7} \left(e^{[Na^+]_o/67300} - 1 \right) \tag{46}$$

$$E_{Na} = \frac{RT}{F} \ln \frac{0.9[Na^+]_o + 0.1[K^+]_o}{0.9[Na^+]_i + 0.1[K^+]_i} \tag{47}$$

$$\frac{d[Na^+]_i}{dt} = -\frac{A_{cap} C_m}{V_{myo} F} (I_{Na} + I_{Nab} + 3I_{NaK} + 3I_{NaCa}) \tag{48}$$

$$I_{Na} = G_{Na}O_{Na}(V - E_{Na}) \tag{49}$$

$$E_{Na} = \frac{RT}{F}\ln\left(\frac{0.9[Na^+]_o + 0.1[K^+]_o}{0.9[Na^+]_i + 0.1[K^+]_i}\right) \tag{50}$$

$$C_{Na3} = 1.0 - (O_{Na} + C_{Na1} + C_{Na2} + IF_{Na} + I1_{Na} + I2_{Na} + IC_{Na2} + IC_{Na3}) \tag{51}$$

$$\frac{dC_{Na2}}{dt} = -(\beta_{Na11}C_{Na2} + \alpha_{Na12}C_{Na2} + \beta_{Na3}C_{Na2}) + \alpha_{Na11}C_{Na3} + \beta_{Na12}C_{Na1} + \alpha_{Na3}IC_{Na2} \tag{52}$$

$$\frac{dC_{Na1}}{dt} = -(\beta_{Na12}C_{Na1} + \alpha_{Na13}C_{Na1} + \beta_{Na3}C_{Na1}) + \alpha_{Na12}C_{Na2} + \beta_{Na13}O_{Na} + \alpha_{Na3}IF_{Na} \tag{53}$$

$$\frac{dO_{Na}}{dt} = -(\beta_{Na13}O_{Na} + \alpha_{Na2}O_{Na}) + \alpha_{Na13}C_{Na1} + \beta_{Na2}IF_{Na} \tag{54}$$

$$\frac{dIF_{Na}}{dt} = -(\beta_{Na2}IF_{Na} + \alpha_{Na3}IF_{Na} + \alpha_{Na4}IF_{Na} + \beta_{Na12}IF_{Na}) + \alpha_{Na2}O_{Na} + \beta_{Na3}C_{Na1} + \beta_{Na4}I1_{Na} + \alpha_{Na12}IC_{Na2} \tag{55}$$

$$\frac{dI1_{Na}}{dt} = -(\beta_{Na4}I1_{N}a + \alpha_{Na5}I1_{Na}) + \alpha_{Na4}IF_{Na} + \beta_{Na5}I2_{Na} \tag{56}$$

$$\frac{dI2_{Na}}{dt} = \alpha_{Na5}I1_{Na} - \beta_{Na5}I2_{Na} \tag{57}$$

$$\frac{dIC_{Na2}}{dt} = -(\beta_{Na11}IC_{Na2} + \alpha_{Na12}IC_{Na2} + \alpha_{Na3}IC_{Na2}) + \alpha_{Na11}IC_{Na3} + \beta_{Na12}IF_{Na} + \beta_{Na13}IC_{Na2} \tag{58}$$

$$\frac{dIC_{Na3}}{dt} = -(\alpha_{Na11}IC_{Na3} + \alpha_{Na3}IC_{Na3}) + \beta_{Na11}IC_{Na2} + \beta_{Na3}C_{Na3} \tag{59}$$

$$\alpha_{Na11} = \frac{3.802}{0.1027e^{-(V+2.5)/17} + 0.2e^{-(V+2.5)/150}} \tag{60}$$

$$\alpha_{Na12} = \frac{3.802}{0.1027e^{-(V+2.5)/15} + 0.23e^{-(V+2.5)/150}} \tag{61}$$

$$\alpha_{Na13} = \frac{3.802}{0.1027e^{-(V+2.5)/12} + 0.25e^{-(V+2.5)/150}} \tag{62}$$

$$\beta_{Na11} = 0.1917\, e^{-(V+2.5)/20.3} \quad (63)$$

$$\beta_{Na12} = 0.2\, e^{-(V-2.5)/20.3} \quad (64)$$

$$\beta_{Na13} = 0.22\, e^{-(V-7.5)/20.3} \quad (65)$$

$$\alpha_{Na3} = 7.0 \times 10^{-7} e^{-(V+7)/7.7} \quad (66)$$

$$\beta_{Na3} = 0.0084 + 2.0 \times 10^{-5}(V + 7.0) \quad (67)$$

$$\alpha_{Na2} = \frac{1.0}{0.393956 + 0.188495\, e^{(V+7.0)/16.6}} \quad (68)$$

$$\beta_{Na2} = \alpha_{Na13}\, \alpha_{Na2}\, \alpha_{Na3}/(\beta_{Na13}\, \beta_{Na3}) \quad (69)$$

$$\alpha_{Na4} = 0.001\alpha_{Na2} \quad (70)$$

$$\beta_{Na4} = \alpha_{Na3} \quad (71)$$

$$\alpha_{Na5} = \alpha_{Na2}/95000 \quad (72)$$

$$\beta_{Na5} = 0.02\alpha_{Na3} \quad (73)$$

$$I_{Nab} = G_{Nab}(V - E_{Na}) \quad (74)$$

$$E_{CaN} = \frac{RT}{2F} \ln \frac{[Ca^{2+}]_o}{[Ca^{2+}]_i} \quad (75)$$

$$I_{CaL} = G_{CaL} O(V - E_{CaL}) \quad (76)$$

$$\frac{d[Ca^{2+}]_i}{dt} = B_i \{J_{leak} + J_{xfer} - J_{up} - J_{trpn} - \left(I_{Cab} + I_{pCa} - 2I_{NaCa}\right) \frac{A_{cap} C_m}{2V_{myo} F}\} \quad (77)$$

$$B_i = \{1 + \frac{[CMDN]_{tot} K_m^{CMDN}}{K_m^{CMDN} + [Ca^{2+}]_i^2}\}^{-1} \quad (78)$$

$$B_{ss} = \{1 + \frac{[CMDN]_{tot} K_m^{CMDN}}{K_m^{CMDN} + [Ca^{2+}]_{ss}^2}\}^{-1} \quad (79)$$

$$B_{JSR} = \{1 + \frac{[CSQN]_{tot}K_m^{CSQN}}{K_m^{CSQN} + [Ca^{2+}]_{JSR}^2}\}^{-1} \tag{80}$$

$$\frac{d[Ca^{2+}]_{ss}}{dt} = B_{ss}\left(J_{rel}\frac{V_{JSR}}{V_{ss}} - J_{xfer}\frac{V_{myo}}{V_{ss}} - I_{CaL}\frac{A_{cap}C_m}{2V_{ss}F}\right) \tag{81}$$

$$\frac{d[Ca^{2+}]_{JSR}}{dt} = B_{JSR}(J_{tr} - J_{rel}) \tag{82}$$

$$\frac{d[Ca^{2+}]_{NSR}}{dt} = \left(J_{up} - J_{leak}\right)\frac{V_{myo}}{V_{NSR}} - J_{tr}\frac{V_{JSR}}{V_{NSR}} \tag{83}$$

$$J_{rel} = \nu_1(O_1 + O_2)\left([Ca^{2+}]_{JSR} - [Ca^{2+}]_{ss}\right)P_{RyR} \tag{84}$$

$$J_{leak} = \nu_2([Ca^{2+}]_{NSR} - [Ca^{2+}]_i) \tag{85}$$

$$J_{xfer} = \frac{[Ca^{2+}]_{ss} - [Ca^{2+}]_i}{\tau_{xfer}} \tag{86}$$

$$J_{up} = \nu_3\frac{[Ca^{2+}]_i^2}{K_{m,up}^2 + [Ca^{2+}]_i^2} \tag{87}$$

$$J_{tr} = \frac{[Ca^{2+}]_{NSR} - [Ca^{2+}]_{JSR}}{\tau_{tr}} \tag{88}$$

$$\begin{gathered} J_{trpn} = -\left(k_{htrpn}^-[HTRPNCa] + k_{ltrpn}^-[LTRPNCa]\right) + \\ k_{htrpn}^+[Ca^{2+}]_i([HTRPN]_{tot} - [HTRPNCa]) \\ +k_{ltrpn}^+[Ca^{2+}]_i([LTRPN]_{tot} - [LTRPNCa]) \end{gathered} \tag{89}$$

$$\frac{dP_{RyR}}{dt} = -0.04P_{RyR} - 0.1\frac{I_{CaL}}{I_{CaL,max}}e^{-\frac{(V-5.0)^2}{648}} \tag{90}$$

$$\frac{d[LTRPNCa]}{dt} = k_{ltrpn}^+[Ca^{2+}]_i([LTRPN]_{tot} - [LTRPNCa]) - k_{ltrpn}^-[LTRPNCa] \tag{91}$$

$$\frac{d[HTRPNCa]}{dt} = k_{htrpn}^+[Ca^{2+}]_i([HTRPN]_{tot} - [HTRPNCa]) - k_{htrpn}^-[HTRPNCa] \tag{92}$$

$$\frac{dO_1}{dt} = -(k_a^-O_1 + k_b^+[Ca^{2+}]_{ss}^mO_1 + k_c^+O_1) +$$

$$k_a^+[Ca^{2+}]_{ss}^n P_{C1} + k_b^- O_2 + k_c^- P_{C2} \tag{93}$$

$$\frac{dO_2}{dt} = k_b^+[Ca^{2+}]_{ss}^m O_1 - k_b^- O_2 \tag{94}$$

$$\frac{dP_{C2}}{dt} = k_c^+ O_1 - k_c^- P_{C2} \tag{95}$$

$$P_{C1} = 1 - (P_{C2} + O_1 + O_2) \tag{96}$$

$$\frac{dO}{dt} = -(4\beta O + \gamma O + \alpha C_4) + K_{pcb} I_1 + 0.001\left(\alpha I_2 - K_{pcf} O\right) \tag{97}$$

$$\frac{dC_2}{dt} = -(\beta C_2 + 3\alpha C_2) + 4\alpha C_1 + 2\beta C_3 \tag{98}$$

$$\frac{dC_3}{dt} = -(2\beta C_3 + 2\alpha C_3) + 3\alpha C_2 + 3\beta C_4 \tag{99}$$

$$\frac{dC_4}{dt} = -\left(3\beta C_4 + \alpha C_4 + \gamma K_{pcf} C_4\right) + 2\alpha C_3 + 4\beta O + 0.01\left(4K_{pcb}\beta I_1 - \alpha\gamma C_4\right) + 0.002\left(4\beta I_2 - K_{pcf} C_4\right) + 4\beta K_{pcb} I_3 \tag{100}$$

$$\frac{dI_1}{dt} = -K_{pcb} I_1 + \gamma O + 0.001\left(\alpha I_3 - K_{pcf} I_1\right) + 0.01\left(\alpha\gamma C_4 - 4\beta K_{pcf} I_1\right) \tag{101}$$

$$\frac{dI_2}{dt} = -\gamma I_2 + 0.001\left(K_{pcf} O - \alpha I_2\right) + K_{pcb} I_3 + 0.002\left(K_{pcf} C_4 - 4\beta I_2\right) \tag{102}$$

$$\frac{dI_3}{dt} = -\left(4\beta K_{pcb} I_3 + K_{pcb} I_3\right) + 0.001\left(K_{pcf} I_1 - \alpha I_3\right) + \gamma I_2 + \gamma K_{pcf} C_4 \tag{103}$$

$$\alpha = \frac{0.4 e^{0.1(V+12.0)}\left[1.0 - 0.75\, e^{-0.0025(V+20)^2} + 0.7\, e^{-0.1(V+40)^2}\right]}{1 + 0.12\, e^{0.1(V+12)}} \tag{104}$$

$$\beta = 0.05 e^{-(V+12)/13} \tag{105}$$

$$\gamma = \frac{K_{pc,max}[Ca^{2+}]_{ss}}{K_{pc,half} + [Ca^{2+}]_{ss}} \tag{106}$$

$$K_{pcf} = 13\left[1 - e^{-0.01(V+14.5)^2}\right] \tag{107}$$

$$I_{pCa} = I_{pCa}^{max} \frac{[Ca^{2+}]_i^2}{K_{m,pCa}^2 + [Ca^{2+}]_i^2} \tag{108}$$

$$I_{Cab} = G_{Cab}(V - E_{CaN}) \tag{109}$$

$$I_{NaCa} = k_{NaCa}\left(\frac{1}{K_{m,Na}^3 + [Na^+]_o^3}\right)\left(\frac{1}{K_{m,Ca} + [Ca^{2+}]_o}\right)$$
$$\cdot\left(\frac{1}{1 + k_{sat}e^{(\eta-1)VF/RT}}\right) \times \{e^{\eta VF/RT}[Na^+]_i^3[Ca^{2+}]_o - e^{(\eta-1)VF/RT}[Na^+]_o^3[Ca^{2+}]_i\} \tag{110}$$

$$I_{Cl,Ca} = G_{Cl,Ca}O_{Cl,Ca}(V - E_{Cl})\frac{[Ca^{2+}]_i}{[Ca^{2+}]_i + K_{m,Cl}} \tag{111}$$

$$O_{Cl,Ca} = \frac{0.2}{1 + e^{-(V-46.7)/7.8}} \tag{112}$$

$$t_s = t - t_{start} \tag{113}$$

$$heav(x) = \begin{cases} 0 & x < 0 \\ 1 & x \geq 0 \end{cases} \tag{114}$$

$$r_{star} = r_0 + pulse\left[heav\big(mod(t_s, period) - t_f\big) - heav\left(mod(t_s, period) - \left(t_f + t_p\right)\right)\right] \tag{115}$$

Supplementary Table 4 List of parameters in the model

Parameter	Description	Value
A_{cap}	Capacitive membrane area	$1.534 \times 10^{-4} cm^2$
V_{myo}	Myoplasmic volume	2.584×10^{-5} μl
V_{JSR}	Junctional SR volume	1.2×10^{-7} μl
V_{NSR}	Network SR volume	2.098×10^{-6} μl
V_{ss}	Subspace volume	1.485×10^{-9} μl
$[Na^+]_i$	Myoplasmic Na^+ concentration	14237.1 μM
$[K^+]_i$	Myoplasmic K^+concentration	143720.0 μM
$[Ca^{2+}]_i$	Myoplasmic Ca^{2+} concentration	0.115001 μM
$[K^+]_o$	Extracellular K^+ concentration	5400.0 μM
$[Na^+]_o$	Extracellular Na^+ concentration	140000.0 μM
$[Ca^{2+}]_o$	Myoplasmic Ca^{2+} concentration	1800.0 μM
$[Ca^{2+}]_{ss}$	Subspace SR Ca^{2+} concentration	0.115001 μM
$[Ca^{2+}]_{JSR}$	JSR Ca^{2+} concentration	1299.50 μM
$[Ca^{2+}]_{NSR}$	NSR Ca^{2+} concentration	1299.50 μM
$[LTRPNCa]$	Concentration Ca^{2+} bound low-affinity troponin-binding sites	11.2684 μM
$[HTRPNCa]$	Concentration Ca^{2+} bound high-affinity troponin-binding sites	125.290 μM
$[LTRPN]_{tot}$	Total myoplasmic troponin low-affinity site concentration	70.0 μM
$[HTRPN]_{tot}$	Total myoplasmic troponin high-affinity site concentration	140.0 μM
C_m	Specific membrane capacitance	1.0 μF/cm^2
F	Faraday constant	96.5 $C/mmol$
T	Absolute temperature	298 K
R	Ideal gas constant	$8.314 J \cdot mol^{-1} \cdot K^{-1}$
$E_{Ca,L}$	Reversal potential for L-type Ca^{2+} channel	63.0 mV
E_{Cl}	Reversal potential for Ca^{2+}-activated Cl^- current	-40.0 mV
k_{NaCa}	Scaling factor of Na^+/Ca^{2+} exchage	992.8 pA/pF
$K_{m,Na}$	Na^+ half-saturation constant for Na^+/Ca^{2+} exchange	87500.0 μM
$K_{m,Ca}$	Ca^{2+} half-saturation constant for Na^+/Ca^{2+} exchange	1380 μM
k_{sat}	Na^+/Ca^{2+}exchange saturation factor at very negative potentials	0.1
η	Controls voltage dependence of Na^+/Ca^{2+} exchange	0.35
I_{NaK}^{max}	Maximum Na^+/K^+ exchange current	0.88 pA/pF
$K_{m,Nai}$	Na^+ half-saturation constant for Na^+/K^+exchange current	21.0 mM
$K_{m,Ko}$	K^+ half-saturation constant for Na^+/K^+ exchange current	1.5 mM
I_{pCa}^{max}	Maximum Ca^{2+} pump current	1.0 pA/pF
$K_{m,pCa}$	Ca^{2+} half-saturation constant for Ca^{2+} pump current	0.5 μM
G_{Na}	Maximum fast Na^+ current conductance	13 $mS/$μF
G_{CaL}	Specific maximum conductivity for L-type Ca^{2+} channel	1.51729 $mS/$μF
G_{Ks}	Maximum slow delayed-rectifier K^+current conductance	0.000575 $mS/$μF
G_{Kr}	Maximum rapid delayed-rectifier K^+ current conductance	0.0078 $mS/$μF
G_{Kur}	Maximum ultrarapidly delayed-rectifier K^+ current conductance(apex)	0.0016 $mS/$μF
$G_{Cl,Ca}$	Maximum Ca^{2+}-activated Cl^- current conductance	10.0 $mS/$μF
G_{Kss}	Maximum noninactivating steady-state K^+ current conductance (apex)	0.05 $mS/$μF
G_{Cab}	Maximum background Ca^{2+} current conductance	3.67×10^{-4} $mS/$μF
G_{Nab}	Maximum background Na^+ current conductance	0.0026 $mS/$μF
$G_{Kto,f}$	Maximum transient outward K^+ current conductance (apex)	0.4067 $mS/$μF
$G_{Kto,s}$	Maximum transient outward K^+ current conductance (apex)	0.01 $mS/$μF
$K_{pc,max}$	Maximum time constant for Ca^{2+}-induced inactivation	0.23324 ms^{-1}
$K_{pc,half}$	Half-saturation constant for Ca^{2+}-induced inactivation	20.0 μM
K_{pcb}	Voltage-insensitive rate constant for inactivation	5.0×10^{-4} ms^{-1}

$I_{CaL,max}$	Normalization constant for L-type Ca^{2+} current	$7.0\, pA/pF$
ν_1	Maximum RyR channel Ca^{2+} permeability	$4.5\, ms^{-1}$
ν_2	Ca^{2+} leak rate constant from the NSR	$1.7 \times 10^{-5}\, ms^{-1}$
ν_3	SR Ca^{2+}-ATPase maximum pump rate	$0.45\, \mu M/ms$
$K_{m,up}$	Half-saturation constant for SR Ca^{2+}-ATPase pump	$0.5\, \mu M$
τ_{xfer}	Time constant for transfer from subspace to myoplasm	$8.0\, ms$
τ_{tr}	Time constant for transfer from NSR to JSR	$20.0\, ms$
k_a^+	RyR P_{C1} - P_{O1} rate constant	$0.006075\, \mu M^{-4}/ms$
k_b^+	RyR P_{O1} - P_{O2} rate constant	$0.00405\, \mu M^{-3}/ms$
k_a^-	RyR P_{O1} - P_{C1} rate constant	$0.07125\, ms^{-1}$
k_b^-	RyR P_{O2} - P_{O1} rate constant	$0.965\, ms^{-1}$
k_c^-	RyR P_{C2} - P_{O1} rate constant	$8.0 \times 10^{-4}\, ms^{-1}$
k_c^+	RyR P_{O1} - P_{C2} rate constant	$0.0090\, ms^{-1}$
n	RyR Ca^{2+} cooperativity parameter P_{C1} - P_{O1}	4.0
m	RyR Ca^{2+} cooperativity parameter P_{O1} - P_{O2}	3.0
$[LTRPN]_{tot}$	Total myoplasmic troponin low-affinity site concentration	$70.0\, \mu M$
$[HTRPN]_{tot}$	Total myoplasmic troponin high-affinity site concentration	$140.0\, \mu M$
$[CSQN]_{tot}$	Total junctional SR calsequestrin concentration	$15000.0\, \mu M$
$[CMDN]_{tot}$	Total myoplasmic calmodulin concentration	$50.0\, \mu M$
$[LTRPNCa]_{tot}$	Concentration Ca^{2+} bound low-affinity troponin-binding sites	$11.2684\, \mu M$
$[HTRPNCa]_{tot}$	Concentration Ca^{2+} bound high-affinity troponin-binding sites	$125.29\, \mu M$
k_{ltrpn}^+	Ca^{2+} on rate constant for troponin low affinity sites	$0.0327\, \mu M^{-1}/ms$
k_{htrpn}^+	Ca^{2+} on rate constant for troponin high affinity sites	$0.00237\, \mu M^{-1}/ms$
k_{ltrpn}^-	Ca^{2+} off rate constant for troponin low affinity sites	$0.196\, ms^{-1}$
k_{htrpn}^-	Ca^{2+} off rate constant for troponin high affinity sites	$3.2 \times 10^{-5}\, ms^{-1}$
K_m^{CMDN}	Ca^{2+} half-saturation constant for calmodulin	$0.238\, \mu M$
K_m^{CSQN}	Ca^{2+} half-saturation constant for calsequestrin	$800.0\, \mu M$
k_f	Rate constant for rapid delayed-rectifier K^+ current	$0.023761\, ms^{-1}$
k_b	Rate constant for rapid delayed-rectifier K^+ current	$0.036778\, ms^{-1}$
$K_{m,Cl}$	Half-saturation constant for Ca^{2+}-activated Cl^- current	$10.0\, \mu M$
O	L-type Ca^{2+} channel conducting state	9.30308×10^{-19}
O_1	Fraction of RyR channels in state O_1	1.49102×10^{-5}
O_2	Fraction of RyR channels in state O_2	9.51726×10^{-11}
C_1	L-type Ca^{2+} channel closed state	0.999876
C_2	L-type Ca^{2+} channel closed state	1.24216×10^{-4}
C_3	L-type Ca^{2+} channel closed state	5.78679×10^{-9}
C_4	L-type Ca^{2+} channel closed state	1.19816×10^{-13}
I_1	L-type Ca^{2+} channel inactivated state	4.97023×10^{-19}
I_2	L-type Ca^{2+} channel inactivated state	3.45847×10^{-14}
I_3	L-type Ca^{2+} channel inactivated state	1.85106×10^{-14}
P_{C1}	Fraction of RyR channels in state P_{C1}	0.999817
P_{C2}	Fraction of RyR channels in state P_{C2}	1.6774×10^{-4}
P_{RyR}	RyR modulation factor	0.0
C_{Na1}	Closed state of fast Na^+ channel	2.79132×10^{-4}
C_{Na2}	Closed state of fast Na^+ channel	0.020752
C_{Na3}	Closed state of fast Na^+ channel	0.624646
O_{Na}	Closed state of fast Na^+ channel	7.13483×10^{-7}
IF_{Na}	Fast inactivated state of fast Na^+ channel	1.53176×10^{-4}
$I1_{Na}$	Slow inactivated state 1 of fast Na^+ channel	6.73345×10^{-7}
$I2_{Na}$	Slow inactivated state 2 of fast Na^+ channel	1.55787×10^{-9}
IC_{Na2}	Closed-inactivated state of fast Na^+ channel	0.0113879
IC_{Na3}	Closed-inactivated state of fast Na^+ channel	0.34278
$a_{to,f}$	Gating variable for transient outward K^+ current	0.00265563

$i_{to,f}$	Gating variable for transient outward K^+ current	0.999977
$a_{to,s}$	Gating variable for transient outward K^+ current	4.17069×10^{-4}
$i_{to,s}$	Gating variable for transient outward K^+ current	0.998543
n_{Ks}	Gating variable for slow delayed-rectifier K^+ current	2.62753×10^{-4}
a_{ur}	Gating variable for ultra rapidly activating delayed-rectifier K^+ current	4.17069×10^{-4}
i_{ur}	Gating variable for ultra rapidly activating delayed-rectifier K^+ current	0.998543
a_{Kss}	Gating variable for noninactivating steady-state K^+ current	4.17069×10^{-4}
i_{Kss}	Gating variable for noninactivating steady-state K^+ current	1
C_{K0}	mERG channel closed state	0.998159
C_{K1}	mERG channel closed state	9.92513×10^{-4}
C_{K2}	mERG channel closed state	6.41229×10^{-4}
O_K	mERG channel open state	1.75298×10^{-4}
I_K	mERG channel inactivated state	3.19129×10^{-5}
V	Membrane potential	$-82.4202\ mV$
t	Time	$0.0\ ms$
r_0	Initial amplitude	$1\, ms$
$period$	Stimulation cycle	$300\, ms$
$pulse$	Stimulation amplitude	$10\, pA/pF$
t_{start}	Initial time lapse	$10\, ms$
